# Molecular Hydrogen Therapy: Mechanisms, Delivery Methods, Preventive, and Therapeutic Application

**DOI:** 10.1002/mco2.70194

**Published:** 2025-04-28

**Authors:** Jiayi Jin, Lijun Yue, Maoru Du, Feng Geng, Xue Gao, Yuming Zhou, Qianqian Lu, Xiaohong Pan

**Affiliations:** ^1^ School of Pharmacy Binzhou Medical University Yantai China; ^2^ Department of Laboratory Medicine Yantai Affiliated Hospital of Binzhou Medical University Yantai China; ^3^ Department of Oncology Yantai Affiliated Hospital of Binzhou Medical University Yantai China

**Keywords:** delivery methods, intestinal diseases, molecular hydrogen (H_2_), mechanisms of action, nanoplatforms

## Abstract

Molecular hydrogen (H_2_), recognized as the smallest gas molecule, is capable of permeating cellular membranes and diffusing throughout the body. Due to its high bioavailability, H_2_ is considered a therapeutic gas for the treatment of various diseases. The therapeutic efficacy of hydrogen is contingent upon factors such as the administration method, duration of contact with diseased tissue, and concentration at targeted sites. H_2_ can be administered exogenously and is also produced endogenously within the intestinal tract. A comprehensive understanding of its delivery mechanisms and modes of action is crucial for advancing hydrogen medicine. This review highlights H₂’s mechanisms of action, summarizes its administration methods, and explores advancements in treating intestinal diseases (e.g., inflammatory bowel disease, intestinal ischemia–reperfusion, colorectal cancer). Additionally, its applications in managing other diseases are discussed. Finally, the challenges associated with its clinical application and potential solutions are explored. We propose that current delivery challenges faced by H_2_ can be effectively addressed through the use of nanoplatforms; furthermore, interactions between hydrogen and gut microbiota may provide insights into its mechanisms for treating intestinal diseases. Future research should explore the synergistic effects of H_2_ in conjunction with conventional therapies and develop personalized treatment plans to achieve precision medicine.

## Graphical abstract

Molecular hydrogen, delivered through inhalation, hydrogen‐rich water, or hydrogen solution injection, reduces oxidative stress, regulates immune responses, facilitates cell repair, and alleviates inflammatory bowel disease (IBD) and intestinal injury. Its protective mechanisms in ischemia–reperfusion injury and colorectal cancer are associated with the regulation of NF‐κB and PI3K/AKT/mTOR signaling pathways.

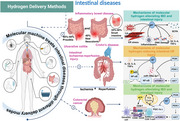

## 1 Introduction

Molecular hydrogen (H_2_) is a colorless, odorless, highly flammable, and poorly soluble gas at normal temperature and pressure. It is also an inert gas with reducing properties [1]. Dole et al. [2] first demonstrated the potential of H_2_ in treating skin cancer in 1975, marking the initial presentation of H_2_ as a therapeutic tool. However, this discovery did not immediately translate into a broader clinical application for H_2_. It was not until 2007 that Ohsawa et al. [3] published an article in *Nature Medicine*, illustrating the selective scavenging of hydroxyl radicals (•OH) and peroxynitrite anion (ONOO⁻ONOO‐) using 2% H_2_ gas to treat cerebral ischemia–reperfusion (I/R) injury in rats. This breakthrough represented a significant advancement in the field of H_2_ medicine. Since then, numerous studies have demonstrated the protective and therapeutic effects of H_2_ in a variety of pathologies, including neurodegenerative diseases [4, 5, 6, 7], cardiovascular disease [8], cancer [9, 10], dermatological conditions [11], sepsis [12], hematological diseases [13], and COVID‐19 [14]. The therapeutic effects of H_2_ in these domains are attributed not only to its antioxidant properties but also to its anti‐inflammatory, antiapoptotic, and antiallergic capabilities. An in‐depth understanding of the mechanisms of action underlying H_2_ is critical for effectively leveraging its potential across different medical conditions.

Unlike other gaseous signaling molecules such as carbon monoxide, nitric oxide (NO), and H_2_ sulfide (H_2_S), H_2_ has an excellent safety profile and exerts protective effects without toxicity risks, even at high concentrations [15, 16]. Despite its high biocompatibility, clinical use of H_2_ is limited by its low solubility and the challenges associated with its high diffusivity [17]. Several studies have demonstrated that H_2_ functions endogenously as a signaling molecule [18], regulating specific molecular pathways [19]. There are two primary strategies for delivering H_2_ for therapeutic purposes in both animal models and human patients: exogenous administration and endogenous production. Exogenous delivery methods include H_2_ inhalation [20], consumption of H_2_‐rich water (HRW) [21], injection of H_2_‐rich saline (HRS) [22], and H_2_ baths [23]. Endogenous H_2_ production can be enhanced by the intake of substances like inulin and lactulose, which promote H_2_ production by intestinal H_2_‐producing bacteria [24]. Selecting the appropriate H_2_ delivery method is critical and should be tailored according to the specific disease being treated.

The gut, as the largest digestive organ in the human body [25], harbors a diverse community of gut microbiota (GM), which plays a pivotal role in the maturation of the intestinal mucosal immune system and maintains a unique regional immune profile [26]. The GM predominantly resides in the lower gastrointestinal tract, specifically within the colon and rectum [27]. Over 100 trillion bacteria are present in the large intestine, approximately 70% of which are H_2_‐producing bacteria capable of producing acetic and butyric acids via hydrogenase (an enzyme that catalyzes H_2_ metabolism and carbohydrate fermentation) [28]. Moreover, the bacterial hydrogenase can utilize exogenous H_2_ to modulate the microbial composition of the gut [29]. Consequently, GM plays a pivotal role in maintaining the health of the digestive system, and dysbiosis is linked to various digestive disorders [30, 31]. Common intestinal diseases include inflammatory bowel disease (IBD), colorectal cancer (CRC), intestinal I/R injury, irritable bowel syndrome (IBS), and intestinal obstruction. Among these ailments, IBD and CRC significantly contribute to global morbidity and mortality rates [32]. IBD is often referred to as “green cancer” due to its incurable and recurring nature [33, 34] and is also associated with an elevated susceptibility to CRC [35], age‐related diseases [36], and other cancers [37]. CRC is the second leading cause of cancer‐related deaths globally, often diagnosed at an advanced stage due to its asymptomatic early course, which makes it challenging to cure. Furthermore, there has been a concerning upward trend in the incidence of CRC among young people [38]. Therefore, it is imperative to explore safe and effective preventive and therapeutic strategies. Given the link between GM and intestinal diseases, H_2_, particularly as an endogenous gas produced by GM, presents itself as a promising therapeutic agent. To advance H_2_‐based therapies, it is crucial to effectively translate findings from animal studies into clinical settings and promote clinical trials. Actively promoting the clinical translation of H_2_ by aggregating and evaluating existing clinical trial data is a key strategy for advancing H_2_‐based therapies.

In this study, we provide a comprehensive analysis of the known biological mechanisms of action of H_2_ and various H_2_ delivery methods, summarizing their advantages and limitations. Furthermore, we review recent advancements in the application of H_2_ for treating intestinal diseases. We also discuss current clinical applications of H_2_ in managing respiratory, neurological, cardiovascular, chronic, and cancer‐related diseases. Last, we highlight the challenges facing the clinical translation of H_2_ and propose potential solutions to overcome these barriers.

## 2 Mechanisms of Molecular H_2_

In the research on the applications of molecular H_2_, a growing body of evidence suggests that molecular H_2_ is not merely a simple antioxidant. Its biological mechanisms are intricate and multifaceted. As research progresses, molecular H_2_ has been found to regulate physiological processes in the body through multiple pathways, exhibiting a wide range of effects such as anti‐inflammatory, antioxidant, regulation of cell apoptosis, improvement of energy metabolism, and modulation of immune function. Therefore, a systematic elucidation of the mechanisms underlying molecular H_2_ is of great significance for understanding its therapeutic potential and clinical applications.

### 2.1 Anti‐Inflammatory Effect

Inflammation is a protective response of the organism to various types of stimuli, including external damage (such as infection and physical injury) and internal imbalances (such as metabolic disturbances and autoimmune reactions) [39]. The process of inflammation resolution can be divided into stages such as the clearance of stimuli, attenuation of proinflammatory signals, removal of inflammatory cells, macrophage phenotypic transformation, and tissue repair [40, 41, 42]. It is important to note that when inappropriate, excessive, or uncontrolled inflammation occurs, it can lead to a range of human diseases, and thus timely suppression of the progression of tissue inflammation and the development of new anti‐inflammatory strategies are necessary. Since the Nakao team [43] first confirmed in 2008 that H_2_ inhalation could improve transplant‐related intestinal injury by inhibiting oxidative stress, numerous studies have subsequently validated the therapeutic potential of molecular H_2_ in acute and chronic inflammatory diseases, marking the official entry of H_2_ therapy into the field of anti‐inflammatory research.

Acute inflammation is the body's direct response to injury or infection, and if acute inflammation is not completely resolved, it can develop into chronic inflammation. The therapeutic effects of H_2_ on both types of acute inflammation have been verified. In the alcohol‐induced liver injury model, H₂ improves liver tissue pathological damage by reshaping the GM homeostasis and inhibiting the activation of the liver Lipopolysaccharide/Toll‐like Receptor 4/Nuclear Factor kappa‐light‐chain‐enhancer of activated B cells （LPS/TLR4/NF‐κB） pathway [44]. In a model of diabetes combined with stroke, H₂ intervention downregulates the expression levels of proinflammatory factors (IL‐1β, IL‐6, Tumor Necrosis Factor‐alpha (TNF‐α)), while activating the TLR4/NF‐κB signaling pathway to achieve neuroprotective effects [45]. In an acute lung injury (ALI) model, H₂ activates the Nrf2 antioxidant pathway and reduces the concentrations of IL‐1β, IL‐6, and TNF‐α, alleviating damage to alveolar epithelial cells [46]. In a sepsis model, H₂ regulates macrophage polarization (inhibiting the M1 phenotype/promoting the M2 phenotype) and inhibits mammalian target of rapamycin (mTOR) phosphorylation, reducing the release of inflammatory mediators such as IL‐6, TNF‐α, and HMGB1, while increasing the levels of anti‐inflammatory factors IL‐10 and Transforming Growth Factor‐beta (TGF‐β) [47]. These findings systematically reveal the multitarget action characteristics of H₂ in the regulation of acute inflammation.

**FIGURE 1:**
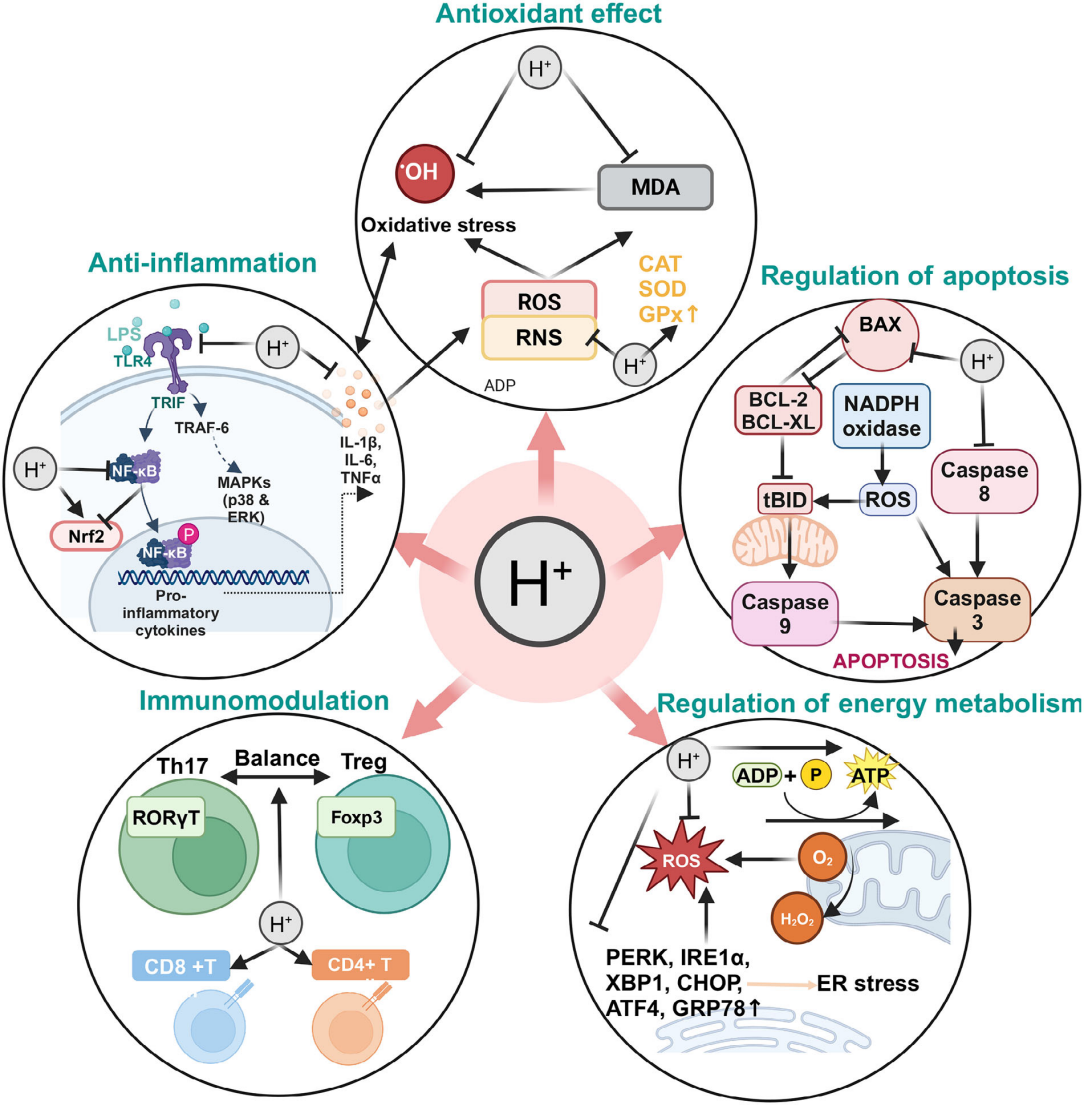
A brief diagram of the mechanisms of molecular hydrogen. The main mechanisms of action of hydrogen are anti‐inflammatory, antioxidant, regulation of apoptosis, regulation of energy metabolism, and immunomodulation. The anti‐inflammatory effect is mainly exerted by inhibiting the secretion of proinflammatory factors and the activation of proinflammatory signaling pathways. The antioxidant effect is mainly achieved by the selective neutralization of •OH and ONOO‐, and inhibition of ROS and MDA production. Regulation of apoptosis is mainly achieved by inhibiting apoptosis in normal cells and promoting apoptosis in cancer cells. Regulation of energy metabolism is mainly achieved by promoting ATP production and inhibiting oxidative stress in the endoplasmic reticulum. The immunomodulatory effects are mainly exerted by maintaining the Th17/Treg balance and promoting the production of CD4+ T and CD8+ T cells.

Due to H_2_’s clear anti‐inflammatory effects and the absence of the severe side effects associated with steroid drugs, it holds greater advantages and application potential in the treatment and prevention of chronic inflammation. It has been found that the activation of NLRP3 plays a role in many chronic inflammatory diseases, and molecular H_2_ can effectively inhibit the activation of NLRP3 to ameliorate endometrial cancer [8], kidney inflammation [48], septicemia [49], neuropathic pain [50], and so on. The anti‐inflammatory mechanism of H₂ mainly focuses on the regulatory network of inflammatory factors (the balance of proinflammatory/anti‐inflammatory factors) and the crosstalk of key signaling pathways (such as NF‐κB, Nrf2, mTOR), which is the current consensus in research [51, 52]. For example, NF‐κB activation facilitates NLRP3 inflammasome assembly, whereas Nrf2 induction suppresses these pathways via redox homeostasis modulation. Notably, sex‐specific differences in patients may influence these mechanisms. An et al. [53] demonstrated that H_2_’s therapeutic efficacy is regulated by sex hormones, and exogenous estrogen supplementation enhances H_2_‐mediated neuroprotection in male mice with intracerebral hemorrhage through inhibition of p‐NF‐κB and p‐IKKβ expression. This finding represents a pivotal consideration for advancing personalized clinical applications of molecular H_2_ in inflammation‐associated disorders (Figure 1).

### 2.2 Antioxidant Effect

Recent research progress has revealed the vicious cycle mechanism between persistent oxidative stress and chronic inflammation—persistent oxidative stress is a major trigger of chronic inflammation, and the occurrence of chronic inflammation increases oxidative stress. Therefore, it is necessary to prevent persistent oxidative stress and select effective and safe antioxidants. Oxidative stress is essentially the dynamic imbalance between the generation rate of reactive oxygen species (ROS)/reactive nitrogen species and the clearance capacity of the endogenous antioxidant defense system. This leads to elevated ROS levels, and extensive interaction between free radicals and biomacromolecules, which in turn causes mitochondrial dysfunction, DNA repair mechanism damage, biomacromolecule oxidation modification, and cell programmed death cascade reactions [54]. The antioxidant effect of H₂ has been studied for 17 years. It differs from antioxidant enzymes (superoxide dismutase [SOD], catalase, and glutathione peroxidase [GPX]) and small‐molecule antioxidants (vitamins C and E, flavonoids, carotenoids, melatonin, ergothioneine, etc.). H₂ has a selective antioxidant effect, meaning it specifically neutralizes the harmful free radicals •OH and ONOO⁻, but does not affect other superoxide anions and peroxides involved in normal physiological processes [3, 55, 56]. Furthermore, H₂ can activate the endogenous antioxidant system by upregulating the expression and activity of Nrf2, a transcription factor that induces the expression of various antioxidant enzymes [57, 58, 59], thereby enhancing the activity of antioxidant enzymes such as SOD, GPX, and glutathione reductase. H₂ mediates ROS regulation through Nrf2, inhibiting NF‐κB/NLRP3 inflammasome activation and achieving an antioxidant–anti‐inflammatory synergistic effect [60].

Based on its unique antioxidant properties, H₂ has demonstrated significant therapeutic potential in various oxidative damage models. Numerous studies have proven that inhalation of H_2_ gas or injection of HRS can be used to treat a range of I/R injuries and other oxidative damage diseases. For example, H_2_ can increase the expression of heme oxygenase‐1 (HO‐1) or activate the phosphatidylinositol‐3‐kinase (PI3K)–Akt signaling pathway to improve liver I/R injury [61, 62]; reduce myeloperoxidase (MPO) activity and IL‐1β/TNF‐α levels to alleviate myocardial injury [63]; inhibit the generation of malondialdehyde (MDA) to reduce valve I/R injury [64]; reduce inflammatory cell infiltration to protect skeletal muscle from I/R injury [65]; suppress oxidative stress mediated by NOX2 and angiotensin II type 1 receptor; and reduce thyroid hormone‐induced myocardial hypertrophy in rats [66], reducing of lipid peroxidation to prevent ALI [67]; H_2_ inhalation reduces serum MDA levels and NOX‐1 expression in lung tissue to prevent pulmonary veno‐occlusive disease [20]; H_2_ alleviates ALI by reducing the levels of MDA in the serum [68], and so on. Notably, oxidative stress and inflammation exhibit bidirectional interaction characteristics in the pathological microenvironment: inflammatory cell infiltration can exacerbate ROS explosion, while oxidative damage products (such as lipid peroxides) can positively regulate proinflammatory signaling cascades. H₂ precisely scavenges toxic free radicals such as •OH, while regulating multiple signaling networks like Nrf2/NF‐κB/NLRP3, achieving a synergistic blockade of the oxidative‐inflammatory cascade (Figure 1).

### 2.3 Regulation of Apoptosis

Apoptosis is a form of programmed cell death that efficiently and systematically removes damaged cells, such as those caused by DNA damage or developmental processes [69]. Apoptosis is closely related to other forms of cell death, such as autophagy, necroptosis, and ferroptosis, but according to current literature, the regulatory effect of H₂ on cell death mainly occurs through bidirectional regulation of the apoptosis pathway. The primary mechanism involves regulating the expression of apoptosis‐related proteins to inhibit apoptosis, thereby protecting cells from damage and maintaining normal tissue function. For example, H₂ upregulates the expression of the antiapoptotic factor Bcl‐2 [70, 71] and downregulates the expression of proapoptotic factors Bax, Caspase‐3, Caspase‐8, and Caspase‐12, thus reducing excessive apoptosis [72, 73, 74]. Additionally, H₂ lowers apoptosis levels by inhibiting excessive activation of PARP‐1 [75]. Unlike other diseases, the antitumor effect of H₂ involves promoting apoptosis. CDK4 and CDK6 control the cell cycle transition from G1 phase to S phase, and inhibiting their expression halts tumor cell proliferation and induces apoptosis directly. Studies have demonstrated that H_2_ inhibits CDK4 and CDK6 to restrict lung cancer progression [76].

In the field of cancer therapy, H₂ demonstrates multidimensional proapoptotic properties. Meng et al. [77] found that H₂ can reverse immune escape in lung cancer cells by inhibiting the expression of CD47 and activating the apoptosis program. Jiang et al. [78] demonstrated that H_2_ promotes apoptosis by downregulating Akt phosphorylation and inhibiting the PI3K signaling pathway in non‐small cell lung cancer. Chu et al. [79] found that inhalation of H_2_ suppresses Hypoxia‐Inducible Factor 1 Alpha Subunit (HIF‐1α)/NF‐κB signaling pathway activation and promotes apoptosis in HeLa cells, counteracting cervical carcinogenesis. This bidirectional regulatory capability allows H₂ to protect normal tissues from excessive apoptosis (such as inflammation‐induced cell death) while selectively inducing apoptosis in tumor cells. This microenvironment‐dependent regulation provides a theoretical basis for the precise application of H₂ in both tissue protection and cancer therapy (Figure 1).

### 2.4 Regulation of Energy Metabolism

Mitochondria are the energy factories of cells, responsible for Adenosine Triphosphate (ATP) production, but also participate in important life activities such as cell differentiation, signal transduction, and apoptosis, and can regulate cell growth and the cell cycle. Mitochondrial dysfunction can lead to cardiovascular disease [80], neurodegenerative disease [81], nonalcoholic fatty liver disease (NAFLD) [82], and many other diseases. Therefore, maintaining normal mitochondrial function is crucial. Dumbuya et al.’s study [83] showed that after treating septic rats with HRS, the decline in mitochondrial membrane potential (MMP) and ATP content was improved. HRS protected mitochondrial membrane ultrastructure from damage to some extent and increased the number of mitochondria [83]. Intraperitoneal injection of HRS can prevent mitochondrial lipid peroxidation, thus preventing excessive apoptosis‐induced tissue damage, protecting mitochondrial structure, increasing the antioxidant potential of mitochondria, and enhancing ATP levels in obstructive jaundice mice [84]. Dong et al.’s research [85] showed that inhaling 2% H₂ can regulate mitochondrial function and dynamics, increase MMP and ATP levels, and enhance mitochondrial activity in the treatment of sepsis‐induced liver injury. Mitochondrial–endoplasmic reticulum (ER) interactions are central to cell metabolism, regulating the exchange of lipids and metabolites between organelles [85]. Cui et al. [86] found that inhalation of high concentrations of H_2_ enhances the expression of mitochondrial membrane proteins MFN2 and mitochondrial biogenesis‐related factors (Peroxisome proliferator‐activated receptor gamma coactivator 1 alpha (PGC‐1α), Nuclear factor E2‐related factor 2 (NRF2), and Mitochondrial transcription factor A (TFAM)), while decreasing dystrophin (DRP1) expression, leading to improved mitochondrial function and attenuation of sepsis‐associated encephalopathy (SAE) in septic mice.

Mitochondria–ER interactions serve as central hubs of cellular metabolism, playing key roles in lipid and metabolite exchange, thereby influencing overall cell function and energy balance [87]. Molecular H_2_ regulates not only mitochondrial energy metabolism but also oxidative stress in the ER. Chen et al. [88] found that ER stress damages the autophagy pathway in septic mice by measuring the expression levels of ER stress‐related proteins (such as PERK, IRE1α, and XBP1). H₂ alleviated inflammation and organ damage by inhibiting ER stress and activating the autophagy pathway in septic mice [88]. Sun et al. [89] found that H_2_‐attenuated ER stress is associated with hyperoxic acute lung injury by reducing the expression of CHOP, GRP78, and XBP1. Guan et al. [73] found that H_2_ could downregulate the expression of CHOP, caspase‐12, and GRP78, while inhibiting p38 and c‐Jun N‐terminal kinase (JNK) phosphorylation, and upregulating the LC3‐II/I ratio in chronic indirect hypoxia‐induced renal injury, suggesting that H_2_ reduces ER stress and activates autophagy by inhibiting oxidative stress and JNK/MAPK activation. Shen et al. [90] demonstrated that HRW prevents IBD in mice by reducing levels of p‐eIF2α, ATF4, XBP1, and CHOP, key proteins in ER stress.

Summarizing current research, H_2_ primarily regulates energy metabolism by acting on mitochondria and the ER. Specifically, H₂ can promote ATP production by enhancing MMP and increasing the activity of mitochondrial respiratory chain complexes. Notably, the functional regulation of the mitochondrial–ER interface may be a key node for H₂’s systemic metabolic regulation, and this discovery provides new research directions for further exploring its cellular protective mechanisms (Figure 1).

### 2.5 Immunomodulation

The immunomodulatory effects of H₂ exhibit multidimensional characteristics, primarily enhancing immunity by protecting immune organs, reducing free radical damage and oxidative stress to immune cells, and regulating immune cell subtypes to protect the integrity of the immune system and alleviate immunosuppression. Since maternal immune dysregulation can induce preterm birth (PTB), Aoki et al. [91] used anti‐CD3 ε to activate effector T cells, leading to PTB and upregulating local and systemic proinflammatory responses to explore the regulatory effects of H_2_ on T cells. H_2_ treatment inhibited several T‐cell effector molecules, such as IFN‐γ, IL‐4, and GZMB, without affecting the expression of regulatory T (Treg) cells. It decreased IL‐26 and IL‐22 (Th17 cell effector cytokines), thereby reducing the rate of PTB caused by T cell activation [91]. A series of studies by Chen's team revealed the immunomodulatory effects of H₂. After inhaling H₂ for 2 weeks, patients with advanced non‐small cell lung cancer showed significant improvement in T‐cell exhaustion. Th cell function returned to normal ranges, and exhausted and senescent cytotoxic T cells gradually decreased to normal levels. The total number of Natural Killer T cells (NKT) and cytotoxic Natural Killer cells (NK) subgroups was higher than the pretreatment percentage. Depleted Vδ2 and Vδ1 cells significantly decreased, while cytotoxic Vδ2 cells significantly increased [92]. Additionally, the total number of γδT cells and NKT subgroups was higher than the pretreatment percentage. In thefollowing year, Chen et al. [93] conducted another study evaluating the effect of inhaling H₂ on the immune function of peripheral blood lymphocyte subgroups in healthy individuals. They unexpectedly found that inhaling high‐flow (nonhigh concentration) H_2_ gas had an inhibitory effect on immune function in healthy humans [93]. Li et al. [94] found that HRS upregulated Tregs cells in rats with brain I/R injury and downregulated the expression of miR‐21, miR‐210, and NF‐κB. Furthermore, Zhao et al. [95] found that HRS can protect against radiation‐induced immune dysfunction by restoring the number of CD4+ T and CD8+ T cells in the spleen. Currently, research on the immunomodulatory functions of H₂ is relatively less explored compared with its anti‐inflammatory and antioxidant capabilities. However, it is undeniable that H_2_ has a beneficial protective effect on the immune system, and further research is needed to explore the specific mechanisms of how H₂ modulates immune functions and the optimal administration methods (Figure 1).

## 3 Delivery Methods of Molecular H_2_

As research into the biological mechanisms of molecular H_2_ progresses, its delivery methods have become an increasingly important focus of study. Different delivery methods not only affect the bioavailability of molecular H_2_ but also determine its efficacy and safety in the treatment of various diseases. Common delivery methods include inhalation, oral administration of HRW, injection of HRS, promotion of endogenous H_2_ production, and nanomaterial‐assisted delivery of molecular H_2_. Each delivery method has unique characteristics, and its effectiveness in treating various disease models can vary.

Inhalation of H_2_ gas facilitates efficient distribution throughout the body and is widely utilized as an adjunctive treatment for both acute and chronic diseases, particularly in the management of pulmonary disorders, neurological diseases, and cancer. In comparison, the consumption of HRW and HRW baths offers greater convenience, making them more suitable for daily health maintenance and the treatment of certain chronic conditions. However, these methods face challenges, such as the rapid dissipation of H_2_ and its limited solubility, highlighting the need for future research aimed at enhancing H_2_ solubility in water. The injection of HRS provides a more precise H_2_ delivery approach, as it rapidly increases H_2_ concentrations in the blood and tissues through intravenous or intraperitoneal injection, with broad applications in the treatment of various acute conditions and inflammatory diseases.

With the increasing understanding of the GM, promoting H_2_ production by the gut microbiome has emerged as a novel H_2_ delivery strategy. By modulating the metabolic activities of the GM, endogenous H_2_ production can be enhanced. This approach is noninvasive and sustainable, positioning it as a promising tool for disease prevention and treatment. The application of nanotechnology has further expanded the potential for H_2_ delivery. Due to their unique physicochemical properties, nanomaterials not only improve H_2_ stability but also enable targeted delivery, showing great promise, particularly in the treatment of cancer and chronic diseases. Although various H_2_ delivery modalities have demonstrated specific advantages across different studies, their interactions and synergistic effects still require in‐depth investigation. Herein, we comprehensively summarize H_2_ delivery approaches and analyze the merits and limitations of each modality.

### 3.1 Inhalation of H_2_

Due to the unique physical properties of H_2_, which fall within the explosive range at concentrations ranging from 4 to 74%, it is essential to specify the concentration of H_2_ for inhalation therapy. Inhalation of 2–4% H_2_ has been chosen in many studies using animal models for disease treatment or clinical treatment to ensure safety [96]. After inhalation, H_2_ rapidly permeates all body regions through alveolar ventilation, including inaccessible areas for macromolecules. Importantly, this intervention does not significantly impact physiological parameters such as blood pressure or interfere with red blood cell oxygen‐carrying capacity, thereby posing no risk of hemotoxicity even at high concentrations [16].

The inhalation of H_2_ through a ventilator, mask, or nasal cannula is primarily used as a therapeutic method or adjunctive measure in clinical practice [97]. Due to the high diffusivity of H_2_, it does not consistently act at the target site. Therefore, direct inhalation of H_2_ is often clinically employed as an adjunctive treatment for patients requiring long‐term hospitalization, such as those with lung and organ problems, diseases of the brain and central nervous system (stroke, Alzheimer's disease [98]), and for cancer treatment and postoperative recovery. In organ transplants, inhalation of H_2_ has been shown to reduce intestinal and lung graft damage and prevent organ inflammation [99]. The approval of H_2_ inhalation therapy as an emergency treatment for patients in cardiopulmonary arrest by Japan under Advanced Medical Care B on December 1, 2016 represents a significant international advancement in the field of H_2_ medicine. In addition, the seventh edition of *Chinese Clinical Guidance for COVID‐19 Pneumonia Diagnosis and Treatment (7th edition)* issued by China National Health Commission recommends the administration of oxygen–H_2_ mixture (33.3% O_2_ and 66.6% H_2_) [100], propelling H_2_ to the forefront of current research into therapeutic medical gases. Using statistical analysis and clinical data, Zeng et al. [101] showed that H_2_/oxygen might be a helpful therapeutic medicinal gas to raise oxygen saturation (SpO_2_) and reduce hospital stays for COVID‐19 patients on average (Table 1).

**TABLE 1:** Delivery of hydrogen and its characteristics.

Hydrogen delivery routes	Advantages and disadvantages	References
Inhalation of H_2_	Simple and easy to implement, but risk of explosion	[96, 99, 100, 101]
Drinking HRW	Low cost, limited efficacy	[103, 104, 105, 106]
HRW bathing	Convenient, safe, and widely used in sport	[107, 108]
Intraperitoneal injection of HRS	Widely used in animal models of intestinal diseases	[117, 118, 119]
Intravenous HRS	Rapid diffusion in the blood	[120, 122, 123]
Local application of HRS	Highly goal‐oriented (Organ preservation fluid, eye drops, rinsing fluid)	[121, 124‐127]
Endogenous hydrogen production	Breath test available	[128, 129, 130, 131, 132, 133, 134, 135, 136, 137]
Nanomaterial‐assisted hydrogen delivery	Targeted therapy	[138, 139, 140, 141, 142, 143, 144, 145, 146, 147, 148, 149, 150, 151, 152]

**FIGURE 2:**
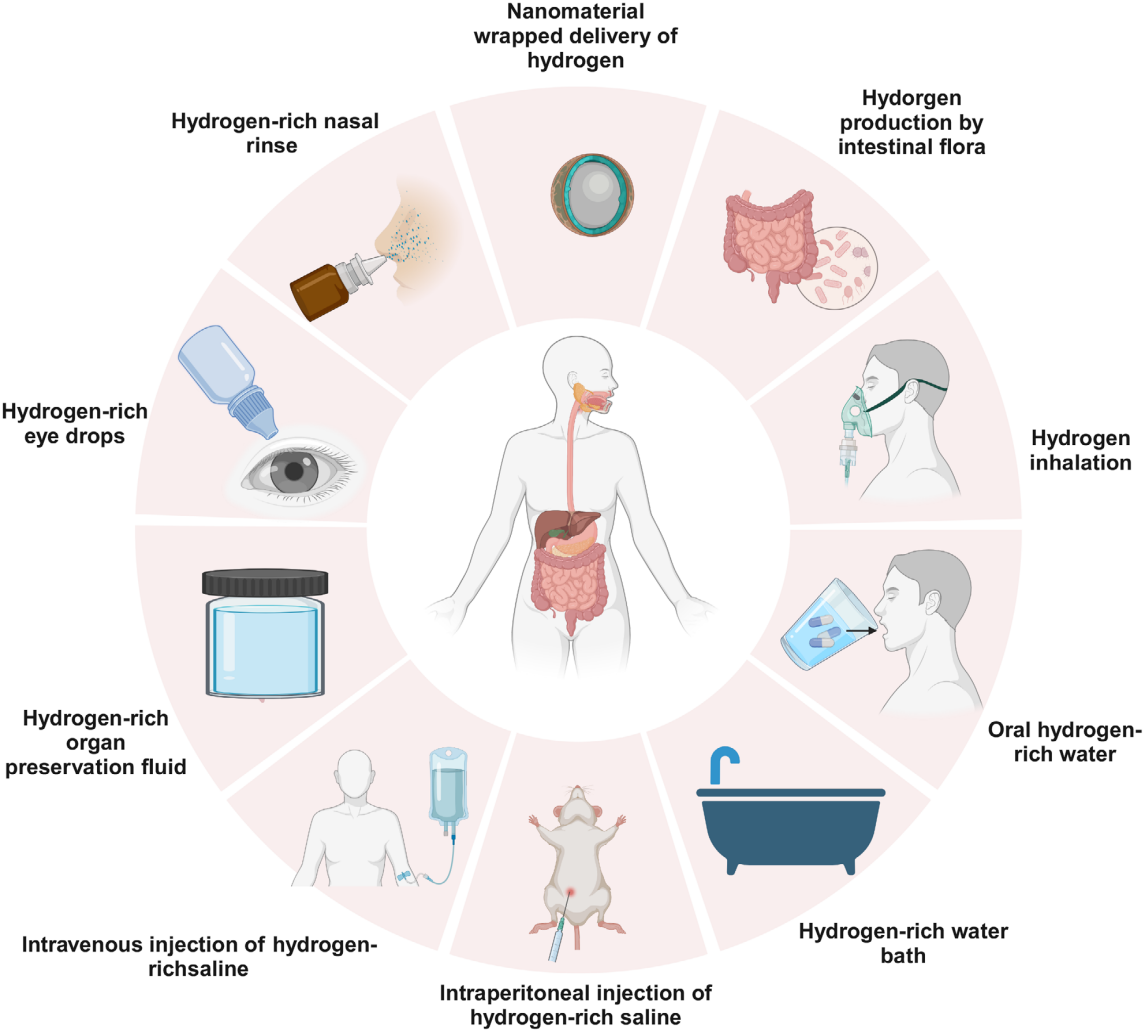
A brief diagram of the hydrogen delivery methods. Hydrogen medicine has evolved to encompass various delivery methods, including endogenous hydrogen production through the intake of lactulose and other substances that promote hydrogen production by gut microbiota (GM). Additionally, exogenous hydrogen can be delivered through inhalation, drinking hydrogen‐rich water (HRW), hydrogen‐enriched baths, injections of hydrogen‐enriched saline, and the use of nanomaterials to assist hydrogen delivery. The exceptional biosafety, anti‐inflammatory, and antioxidant properties of hydrogen have facilitated the development of complementary therapeutic modalities, such as organ preservation fluids enriched with hydrogen, ophthalmic solutions containing high levels of hydrogen, and nasal washes infused with hydrogen.

Inhalation of H_2_ is clinically more feasible and straightforward compared with other methods of H_2_ delivery. Patients can easily self‐administer H_2_ through inhalation, although this method may not be the most convenient. To date, no side effects have been observed with inhaled H_2_ therapy; nevertheless, it necessitates specialized equipment for H_2_ production, rendering its inhalation somewhat inconvenient. The concentration of H_2_ in the blood and tissues depends on the concentration of inhaled H_2_ and the need for continuous inhalation to maintain adequate levels and achieve therapeutic effects. A controversial issue in H_2_ inhalation is the choice between inhalation of a H_2_–oxygen (HO) mixture or pure H_2_, and the dose–effect relationship has not been established. Both HO mixtures and pure H_2_ have their advantages. Because H_2_ has a low molecular weight, inhalation of H_2_ in an HO mixture reduces airway resistance, increases oxygen dispersion, and enhances oxygen flow. At the same time, there is no risk of hypoxia when inhaling the HO mixture, which is usually obtained clinically through water electrolysis (66% H_2_ and 33% oxygen) [62]. Inhalation of HO carries a certain level of risk due to the high partial pressure of H_2_. The presence of oxygen increases the potential for combustion and explosion, especially when the H_2_ concentration exceeds 10%, as it can easily ignite through static electricity without detection, leading to an explosion [33]. Therefore, it is crucial to ensure absolute safety when inhaling HO, often by using H_2_ monitors to continuously monitor its concentration [34]. In comparison, inhalation of pure H_2_ poses a lower risk for humans as it does not cause explosions but carries a higher risk of oxygen deprivation (Figure 2).

### 3.2 Drinking HRW and Taking Baths with HRW

HRW is a low‐cost, portable, and effective way to transfer H_2_. It is safer and more convenient for clinical use compared with inhaled H_2_, as it can be consumed freely while the patient is awake without developing tolerance effects over prolonged usage. Drinking HRW exhibits distinct mechanisms of action and effects when compared with inhaling H_2_. Consumption of HRW is more likely to exert beneficial effects on the gastrointestinal system, as well as on the liver and brain. The potential impact on the brain may also be attributed to the upregulation of entero‐brain axis communication and secondary messenger molecules [102]. The main uptake pathway for drinking HRW is the digestive tract and portal vein system. After entering the pulmonary circulation, most of the ingested H_2_ evaporates from the lungs, leaving a small amount for therapeutic utilization. HRW has been extensively studied in the medical field and has a wide range of applications, but most of the research is currently limited to animal testing, such as its potential for preventing liver damage and improving cognitive disorders [103, 104]. Additionally, there are suggestions that HRW may play a role in regulating endogenous H_2_ homeostasis and modulating GM; however, further trials are needed to explore the possible mechanisms underlying this interaction.

Recent studies have demonstrated that preworkout intake of HRW can effectively reduce lactic acid levels, thereby enhancing ventilation efficiency and exerting an antifatigue effect [105, 106]. In addition to drinking HRW, HRW baths are widely used as a delivery method for HRW. For example, the application of HRW ankle baths has been shown to reduce joint swelling on pain relief to restore mobility and balance, making it a safe and convenient way to address acute ankle sprains in the field of sports medicine [107]. Clinically, HRW baths exhibit inhibitory effects on inflammation and oxidative stress while demonstrating therapeutic benefits for conditions such as psoriasis [108]. Furthermore, through its anti‐inflammatory properties, HRW baths also facilitate the healing process of diabetic foot ulcers [43] (Table 1).

To sum up, HRW holds significant potential for development and application, necessitating further research on its clinical applications in the future. At present, the technology for preparing HRW has reached a certain level of maturity; however, the solubility of H_2_ in water at room temperature and pressure is limited to a maximum of 0.8mM109, resulting in limited efficacy when orally administered. Therefore, enhancing the solubility of hydrogen in water is a crucial issue. The following two methods are commonly used to prepare HRW:

1. Hydrogen pack direct immersion method

The principle of this method is to use the redox reaction of metals to produce HRW. A hydrogen‐producing agent, weighing a total of 0.65g and comprising a mixture of aluminum powder and calcium hydroxide in a weight ratio of 76:2444, is wrapped in a gas‐permeable membrane or non‐woven fabric and directly placed into a polyethylene terephthalate (PET) bottle containing water to generate HRW. The chemical reaction equation for the hydrogen‐producing agent is as follows:

$$2Al^{3+}+\mathrm{Ca}{\left(\mathrm{OH}\right)}_{2}+6H_{2}O\to \mathrm{Ca}{\left[\mathrm{Al}{\left(\mathrm{OH}\right)}_{4}\right]}_{2}+3H_{2}$$

$$Mg^{2+}+2H_{2}O\to \mathrm{Mg}{\left(\mathrm{OH}\right)}_{2}+H_{2}$$

This method is used to produce products such as hydrogen water cups and magnesium sticks110, which enable rapid generation of relatively high concentrations of HRW. However, this process also results in the alkalization of the water and the release of certain metal ions.

2. Electrolysis of water to produce oxygen and hydrogen, the chemical reaction equation is as follows:

$$2H_{2}O\to 2H_{2}+O_{2}$$

Currently, there are two types of electrolytic HRW in the market. One type is based on the traditional method of water electrolysis, where both electrodes are immersed in the water to produce oxygen at the anode and H_2_ at the cathode. This method does not affect the acidity of the water; however, it should be noted that the anode may have a strong oxidizing effect on ions present in the water, potentially leading to ozone and hypochlorite production. The other type utilizes proton permeable membrane technology, which separates the two electrodes to produce alkaline water containing H_2_ called "electrolytic reduced water" at the cathode and acidic oxygen‐containing water at the anode111,112. Although this method of H_2_ production has a long history, it yields low concentrations of H_2_ in the resulting hydrogen‐enriched water (HEW), necessitating urgent efforts to enhance its concentration. With further research and technological improvements, the consumption of HEW could provide an effective means of disease prevention and treatment in the future (Figure 2).

### 3.3 Injection of HRS and Topical Application

The clinical application of H_2_ in hospitals is highly restricted due to safety concerns. Although drinking HRW is generally safe, it is difficult to regulate the amount of H_2_ given because the H_2_ in the water tends to escape over time and some may be lost in the gastrointestinal tract. HRS provides a safer method of H_2_ delivery compared with direct H_2_ inhalation, while also providing better control of H_2_ concentration.

Molecular H_2_ is dissolved into saline at high pressure to produce HRS, a beneficial antioxidant; it has a high H_2_ content, weak alkalinity, and negative potential. Moreover, it has potent anti‐inflammatory, antioxidative stress, and antiapoptotic properties and is safe and nontoxic [113]. HRS is typically administered to the body through intraperitoneal or intravenous injection, and these two different methods of H_2_ delivery elicit distinct changes in tissue H_2_ concentration [114, 115]. Following intraperitoneal injection of HRS, the peak concentrations of H_2_ in blood and various tissues (including liver, kidney, heart, spleen, pancreas, small intestine, muscle, and brain) were observed at 5 min. In contrast, after intravenous injection of HRS, maximum concentration was achieved within 1 min. Both types of injections demonstrated a dose‐dependent increase in H_2_ concentrations within the blood as well as liver, spleen, pancreas, and brain tissue [116]. I/R injury is one of the main global causes of high morbidity, disability, and death rates; therefore, it is imperative to find an efficacious treatment strategy. For example, intraperitoneal injection of HRS has been demonstrated to reduce ALI caused by limb I/R [117] injury, attenuate renal I/R injury in rats [118], and exhibit therapeutic amelioration in a rat model of SAE through the inhibition of NLRP3/Caspase‐1/TLR4 signaling pathway [119]. Additionally, intravenous administration of HRS has shown potential in mitigating intestinal I/R injury in rats [120] while immersion of lungs in HRS has proven effective in reducing pulmonary I/R injury [121]. However, frequent intraperitoneal injections of HRS pose the risk of cross‐infection whereas intravenous administration carries certain dangers [122, 123] (Table 1).

HRS is also widely utilized in the treatment of ocular diseases. It can be injected into the vitreous cavity to reduce retinal excitatory damage and promote recovery of retinal function, or used as a H_2_‐containing eye drop to protect the retina [124, 125]. In addition, a randomized, double‐blind clinical trial demonstrated that HRS nasal rinses were more effective than saline rinses in improving clinical symptoms of chronic rhinitis (CR), particularly in patients with. This highlights the potential effectiveness of HRS in the clinical management of CR patients [126]. Currently, most studies on HRS as an injectable have been conducted in animal trials, and further exploration is needed to promote its clinical application. However, HRS can also be used topically as an organ preservation solution, eye drops, and rinse solution for disease treatment. The common method for producing HRS is to dissolve H_2_ in saline at high pressure (0.4–0.6 MPa) for 2 h to achieve a supersaturation level (>0.6 mmol/L) [127]. Saturated HRS is sterilized using gamma radiation and kept in sealed aluminum bags at 4°C and atmospheric pressure. Freshly HRS is prepared weekly to maintain a constant concentration. Gas chromatography is used to confirm the H_2_ content of saline using the technique outlined by Ohsawa et al. [3], which is widely used for determining H_2_ content in saline (Figure 2).

### 3.4 Endogenous H_2_ Production

Intestinal gases produced by GM include methane (CH_4_), H_2_, H_2_S, and carbon dioxide. Among these gases, H_2_ constitutes up to 74% of the total gases produced by GM. For instance, *Escherichia coli* (*E. coli*) in the intestine can produce H_2_ through its hydrogenase enzyme [128]. However, a significant portion of this H_2_ is used by other bacteria, while a minor fraction is absorbed by the intestinal mucosa and subsequently excreted via pulmonary respiration and defecation. Consequently, the overall H_2_ production of the GM is determined by both its production and consumption. Differences in GM among individuals contribute to variations in H_2_ production, and researchers have conducted GM transplantation experiments across different animals to observe alterations in H_2_ production and differences in disease resistance [129]. However, the amount of H_2_ generated by GM can be easily disturbed by external factors, and the intake of various nondigestible sugars such as high straight‐chain amylose corn starch, pectin, oligofructose, and inulin in mammals can stimulate increased H_2_ production by GM [130, 131]. It has been shown that H_2_ produced by GM exhibits close associations with certain diseases; for example, H_2_ can promote intestinal motility and thus serve as a potential treatment for constipation, while a deficiency of H_2_ may be implicated in the development of Parkinson's disease (PD) [132, 133] (Table 1).

Due to its endogenous nature, H_2_ is being increasingly explored by researchers for breath testing (BT) to achieve rapid diagnosis of clinical gastrointestinal diseases. BT is an effective and noninvasive diagnostic tool for many gastrointestinal disorders [134]. The H_2_ breath test (H_2_BT) works on the basis that part of the gas produced by the fermentation of colonic bacteria diffuses into the abdominal venous circulation and travels to the lungs, where it is easily measured while breathing. Therefore, the H_2_BT has been employed for various purposes: (a) evaluating carbohydrate malabsorption of different absorbable sugars such as lactose and fructose in the small intestine [135], (b) measuring the time interval between ingestion of nonabsorbable carbohydrates (e.g., lactose) and their interaction with bacteria in the cecum and colon (oral‐cecum passage time) [136], and (c) frequently utilizing carbohydrates like glucose or lactulose for H_2_ exhalation to identify small GM overgrowth in IBS (SIBO) [137] (Figure 2).

### 3.5 Nanomaterial‐Assisted H_2_ Delivery

All of these delivery methods, whether exogenous or endogenous H_2_ production, face a common challenge: the accurate delivery of H_2_ to the targeted diseased area. The therapeutic effect of H_2_ would be greatly improved if it could be targeted at the site of disease and released continuously, as well as stored efficiently. Nanomaterials have garnered considerable attention from researchers due to their unique physicochemical properties. Although the clinical translation of nanotechnology remains a significant challenge, it provides a promising platform for the advancement of H_2_ medicine. This has led to the emergence of the concept of nanohydrogen medicine, resulting in the development of numerous nanodelivered H_2_ materials (Table 1).

#### 3.5.1 Nanobubble H_2_ Water

H_2_, being insoluble in water and highly diffusible with greater buoyancy, poses challenges for the production, preparation, and preservation of highly concentrated HRW. Nanobubbles exhibit excellent stability and permeability in liquids, enabling the physical dissolution of H_2_ through nanobubbles to produce nanobubble H_2_ water (NBW). This process allows for maximum dissolution of H_2_ in the water while retaining it for longer periods [138]. Japanese researchers utilized the 2,2′‐bipyridyl method to measure the reducing activity of NBW and confirmed its superior antioxidant activity compared with HRW at equivalent H_2_ concentration [139]. It has been demonstrated that the fine H_2_ nanobubbles are highly stable in water, and there are H_2_ nanobubbles that remain in HRW even after long periods of storage, thereby reducing the rate at which H_2_ escapes from water [140] (Figure 2 and Table 2).

**TABLE 2:** Summary of hydrogen‐carrying nanomaterials.

Hydrogen‐carrying nanomaterials	Characteristics of action	Diseases	References
Treatment of diseases of the locomotor system			
CBN@GelDA hydrogel	Promotion of H_2_ generation and reduction of ROS production	OA	[141]
Mg@PLGA MPs	Can be stored in situ at the injection site	OA	[142]
AB@HPDA	Continuous release of hydrogen	Intervertebral disc degeneration	[143]
Treatment of diseases of the nervous system			
TSIIA/PNS/AB‐loaded CCC@mPP NPs	Exploiting the synergistic effects of multiple drugs	Sensorineural hearing loss	[144]
Treatment of diseases of the endocrine system			
Bacillus‐Chlorella gel patch	Extension of hydrogen delivery time	Diabetic wounds	[145]
Intelligent microneedle patch‐MgH_2_	Promotion of H_2_ generation and reduction of ROS production	Diabetic wounds	[146]
A dual‐action liquid metal dressing	Dual action of antibiotic delivery and hydrogen generation for antibacterial and anti‐inflammatory purposes	Diabetic wounds	[147]
Treatment of diseases of the endocrine system			
C_3_N_4_@Gel	Rapid penetration into the biofilm to achieve an antimicrobial effect	Diabetic wounds	[148]
Treatment of diseases of the circulatory system			
TN‐PdHs	Target‐oriented effect	Atherosclerosis	[149]
PdH nanoparticles	It exerts local capture and storage of H_2_ passing through the liver and rapidly catalyzes the hydrogenation of •OH to H_2_O	Nonalcoholic steatohepatitis	[150]
H_2_‐PFOB nanoemulsions	Ultra‐high biosafety	Myocardial ischemia–reperfusion	[151]
AB@hMSN@PEG	Releases ultra‐high doses of H_2_ in the gut and effectively prolongs the duration of action	Metabolic dysfunction‐associated fatty liver disease	[152]

*Abbreviations*: AB@hMSN@PEG, a novel H_2_ will be more nanocapsule by encapsulating ammonia borane into hollow mesoporous silica nanoparticles; AB@HPDA, ammonia borane‐loaded hollow polydopamine; C_3_N_4_@Gel, C_3_N_4_ nanosheets loading hydrogel; CBN@GelDA hydrogel, an injectable calcium boride nanosheets loaded hydrogel platform; Mg@PLGA MPs, comprising magnesium‐containing poly (lactic acid)‐poly (glycolic acid) particles; OA, osteoarthritis; perfluorooctyl bromide nanoemulsions (PFOB NEs) were synthesized and then hydrogenated by hydrogen absorption; TN‐PdHs, tetrapod needle‐like Pd nanocrystals for H_2_ adsorption.

#### 3.5.2 Nanomaterials as Carriers of H_2_ for the Treatment of Motor and Neurological Disorders

Diseases of the locomotor system are characterized by their insidious onset, slow progression, prolonged duration, and challenging eradication. Therefore, this type of disease is often treated with a combination of drugs, and several studies have demonstrated that the combination of H_2_ and nanoparticles performs well in the treatment of locomotor system diseases. Zhang et al. [141] developed an injectable hydrogel platform loaded with **calcium boride nanosheets** (CBN@GelDA hydrogel), which serves as a highly efficient loaded and sustainably releasable H_2_ precursor for use in osteoarthritis (OA) therapy. This platform effectively scavenges ROS, diminishes the expression of pertinent inflammatory cytokines, and suppresses M1 macrophages while inducing the M2 phenotype. These actions ultimately lower chondrocyte death and aid in breaking the vicious cycle of OA progression [141]. Wan et al. [142] proposed a local drug delivery system comprising magnesium‐containing poly (lactic acid)–poly (glycolic acid) particles (Mg@PLGA MPs) to deliver high concentrations of therapeutic gaseous H_2_ to inflamed tissues. In a mouse model, Mg@PLGA MPs were intramuscularly injected near the knee joints of OA, serving as an in situ reservoir that continuously released a concentration of gaseous H_2_ above the therapeutic threshold. This approach effectively reduced tissue inflammation, prevented cartilage destruction, and halted the progression of OA lesions [142]. Wang et al. [143] developed ammonia borane‐loaded hollow polydopamine (AB@HPDA) to achieve prolonged H_2_ release for the treatment of intervertebral disc degeneration (Table 2).

Previous studies have shown positive results when using H_2_ for the treatment of neurological disorders. Xiao et al. [144] constructed Tanshinone IIA /Panax notoginseng saponins/Ammonia borane /Carboxymethyl chitosan/calcium carbonate‐chitosan NPs/Monomethoxy poly(ethylene glycol)‐PLGA NPs (TSIIA/PNS/AB‐loaded CCC@mPP nanoparticles) that can exert a synergistic polypharmacy effect, indirectly or directly promoting the scavenging of excessive intracellular ROS, reducing the release of inflammatory cytokines, and enhancing the efficacy of traditional Chinese medicine complexes and H_2_ donors in treating sensorineural hearing loss (Table 2).

#### 3.5.3 Nanomaterials as Carriers of H_2_ for the Treatment of Diabetic Wounds

Diabetic wounds are one of the most serious complications of diabetes, posing a significant threat to the lives and health of diabetic patients. However, there are currently limited clinical treatments available for this disease. Molecular H_2_ therapy may be a promising approach to address this issue. Bacillus‐Chlorella (Bac‐Chl) gel patch developed by Chen et al. [145] achieves continuous H_2_ production for 60 h to promote chronic diabetic wound healing in vivo by delivering H_2_ to reduce oxidative stress and inflammation. Wang et al. [146] devised an intelligent microneedle patch‐MgH_2_ capable of prolonged release of MgH_2_ to generate more H_2_, which effectively reduces ROS production and promotes diabetic wound healing. Bi et al. [147] developed a dual‐action liquid metal dressing with antibiotic delivery and H_2_ generation for antimicrobial and anti‐inflammatory purposes, providing a gentle method of H_2_ therapy and drug delivery while treating chronic diabetic wounds. Xu et al. [148] proposed the concept of sonocatalytic H_2_/hole‐combined therapy for antibiofilm and infected diabetic wound healing, developing a piezoelectric C_3_N_4_ nanosheets loading hydrogel (C_3_N_4_@Gel) that produces H_2_ capable of rapidly penetrating biofilms, which is not achievable with conventional antimicrobial agents. This approach demonstrates both antimicrobial activity and promotion of diabetic wound healing through H_2_ release [148]. However, most studies on H_2_ nanomedicine in diabetic wounds have primarily focused on animal experiments, with fewer investigations into the underlying mechanisms and limited clinical evidence to support these findings; thus, further comprehensive research is needed in the future (Table 2).

#### 3.5.4 Nanomaterials as Carriers of H_2_ for the Treatment of Cardiovascular and Liver Diseases

The therapy of liver and cardiovascular illnesses has made substantial use of H_2_. However, the majority of research that has already been done focuses mostly on how H_2_ helps to reduce oxidative damage, inflammation, and apoptosis. There is an urgent need to surpass the current international research levels by exploring novel approaches. Nanohydrogen medicine may offer a new direction. Hu et al. [149] utilized TN‐PdHs for targeted management of atherosclerosis, while Tao et al. [150] intravenously injected PdH nanoparticles into mild and moderate nonalcoholic steatohepatitis (NASH) model mice, followed by daily inhalation of 4% H_2_ for 3 h. The experimental results demonstrated that after the intravenous injection, Pd nanoparticles could be specifically accumulated in the liver and play a dual role as a H_2_ captor and ·OH filter. This enhanced the effectiveness of H_2_ treatment in preventing and treating NASH by enabling them to quickly catalyze the hydrogenation of ·OH to H_2_O and locally capture and store H_2_ that was entering the liver during daily H_2_ inhalation [150]. Nie et al. [151] developed H_2_‐PFOB nanoemulsions (NEs) with high H_2_‐carrying capacity, which effectively facilitate targeted penetration into the ischemic myocardium and exhibit excellent antioxidant and anti‐inflammatory properties against myocardial I/R injury with a good biosafety profile. Jin et al. [152] combined H_2_ with nanomaterials to construct H_2_ nanocapsules AB@hMSN@PEG by encapsulating ammonia borane into hollow mesoporous silica nanoparticles, enabling the release of ultra‐high doses of H_2_ in the intestines. This novel strategy successfully extended the duration of action, consequently reducing the early stages of obesity, diabetes mellitus, and fatty liver disease in mice that are caused by genetic mutations and diet‐induced metabolic dysfunction without generating any tissue toxicity. Furthermore, it increased the abundance of *Akkermansia muciniphila* in the intestinal tract [152]. These findings provide further evidence that H_2_ can exert ameliorative and therapeutic effects on diseases through modulation of the liver–gut axis, highlighting GM remodeling as a potential mechanism for H_2_ treatment of metabolic dysfunction (Table 2).

#### 3.5.5 Nanomaterials Combine with H_2_ for Targeted Tumor Therapy

The combination of nanotechnology and H_2_ therapy can enhance the targeted delivery of H_2_ to tumors, where H_2_ is produced in response to endogenous or external stimuli, thereby maximizing its bioavailability over time. Nanomaterials have three significant advantages: minimal invasiveness, implantability within the body, and rapid biochemical reactivity. Various nanocatalysts such as palladium hydride nanocrystals, PdH‐Metal‐Organic Framework (MOF), Mg‐based galvanic cell, SnS1.68‐WO2.41 nanocatalysts, a kind of biocompatible carboxymethyl cellulose‐coated/stabilized Fe (Fe@CMC) nanoparticle with photoacoustic imaging (PAI), as well as small‐sized nanoparticles (20–100 nm) like Fe and Au–TiO_2_ heterojunction nanoplatforms (AuTiO_2_@ZnS), have been utilized for tumor‐targeted transport in H_2_ therapy [153, 154, 155].

In addition to nanomaterials for H_2_ delivery, H_2_ therapy can serve as a complementary approach to conventional treatments by the energy‐modulating effects of H_2_ to enhance efficacy and reduce toxicity. For instance, continuous administration of H_2_ during cryopreservation has been shown to improve the viability of allogenic osteochondral tissue [156]. Furthermore, novel oral acid‐controlled release H_2_ MgB2@Polyvinylpyrrolidone (PVP) pills have been developed for combined use with injectable adriamycin chemotherapy drugs, aiming at achieving enhanced effectiveness and reduced toxicity in gastric cancer chemotherapy [157, 158]. The biocompatible micromotor platform Mg/PLGA/CHI, developed by Song et al. [159], generates H_2_ during its action to eliminate excess ROS in HepG2 cells and significantly enhances the diffusion of chemotherapeutic drugs (Table 2). In summary, the efficacy of H_2_ delivery via nanomaterials primarily relies on (a) the effective encapsulation of H_2_, (b) the successful delivery of H_2_ to the target area of the body, and (c) the successful release of H_2_.

Each mode of H_2_ delivery has its advantages, and selecting the appropriate mode for a specific disease is critical. While current research primarily focuses on exogenous H_2_, it is essential to explore safe and effective methods to enhance endogenous H_2_ production by GM for disease prevention and treatment. If certain ingredients can be identified as both safe and capable of augmenting endogenous H_2_ production, they may play a positive role in future disease prevention and treatment strategies. The combination of exogenous and endogenous H_2_ may yield unexpected effects in treating certain diseases due to variations in absorption pathways within the body.

## 4 Preventive and Therapeutic Applications for Intestinal Diseases

According to a summary of published articles, H_2_ has been found to invariably involve the gut flora in the treatment of different systemic diseases, for example, inhalation of H_2_ modulates the gut flora to ameliorate acute alcoholic liver injury [160], l‐Arabinose elicits gut‐derived H_2_ production and ameliorates metabolic syndrome in C57BL/6J mice on a high‐fat diet (HFD) [161]. Molecular H_2_ therapy ameliorates metabolic disturbances and inhibits inflammation in mice with SAE by increasing beneficial bacteria and inhibiting harmful bacteria through actions on the GM, and so on [162]. Therefore, we believe that the interaction between H_2_ and GM should not be ignored, and as H_2_ can act on intestinal flora to improve other diseases, then H_2_ directly treating intestinal diseases may bring better results.

Although H_2_ can be delivered in various ways, the current applications of H_2_ in the treatment of intestinal diseases are almost all exogenous, with only one case of endogenous production of H_2_ by intestinal flora for the treatment of intestinal diseases. According to statistics, the delivery mode of H_2_ varies among different intestinal diseases. The shortest duration of action is 7 days, and the longest is 63 days in the animal model of IBD. For the injection of HRS and the inhalation of H_2_ in the treatment of intestinal I/R injuries, and the inhalation of H_2_ every day or the consumption of HRW in the treatment of CRC. Two studies achieved targeted treatment of intestinal injuries using nanomaterials encapsulating H_2_. In this section, we summarize the effects and mechanisms of H_2_ in the treatment of intestinal diseases, mainly simple bowel injury, IBD, intestinal I/R injury, and CRC.

### 4.1 IBD and Intestinal Injury

IBD, which typically encompasses Crohn's disease and Ulcerative colitis (UC), is characterized by a dysregulated autoimmune response to intestinal ecological dysregulation, triggered by exposure to various irritating environmental factors [163]. The main therapeutic goal for patients with IBD is to reduce inflammation and alleviate symptoms such as abdominal pain and bowel changes. Endoscopy combined with biopsy represents the most efficacious approach for establishing a diagnosis and managing the disease [164]. Current drug treatments for IBD include conventional and biological therapies, with conventional treatments involving anti‐inflammatory drugs, immunosuppressants, antibiotics, and probiotics; while biological therapies primarily consist of various anti‐TNF‐α drugs along with numerous other novel biological agents (e.g., JNK inhibitors) [165]. Due to its high biosafety profile, H_2_ has been investigated as a potential treatment option for IBD and intestinal injuries. Increased intestinal inflammation, LPS production, infection risk, and decreased short‐chain fatty acid (SCFA) content are the main features of IBD pathogenesis. According to our summary of the mechanism of action of H_2_ for the treatment of IBD, it is primarily used to inhibit the production of ROS, reduce the levels of proinflammatory factors, inhibit the activation of relevant inflammatory signaling pathways, and promote the growth of intestinal probiotic bacteria and the metabolites of intestinal flora (SCFAs) through the oral intake of HRW or the injection of HRS, to achieve the goal of treating IBD (Table 3).

**TABLE 3:** Mechanism of action of hydrogen in intestinal diseases.

Diseases	Models	Route and protocol	Mechanisms involved	References
Enteropathy	IND‐induced enteropathy in mice	HRW, oral; Duration: 5 days	ROS↓ Production of SCFA↑	[168]
Radiation‐induced intestinal damage	Whole‐body radiotherapy mice	HRS (10 mL/kg BW), intraperitoneally; Duration: single injection	ROS↓ Mitochondrial apoptosis pathways↓	[171]
Constipation	Constipation modeling by oral administration of loperamide to Sprague–Dawley rats	HRW, oral; Duration: 14 days	SIRT1/Nrf2/ HO‐1↑ ROS↓	[172]
IBD	The rat model of IBD induced by LPS	HRW, oral; Duration: 7 months	Nrf‐2 signaling pathway↑ NF‐κB signaling pathway↓	[169]
IBD	A rat model of IBD induced by colorectal administration of TNBS	HRW, oral; Duration: 14 days	MPO, IL‐1β, IL‐6, TNF‐α, ROS↓	[170]
IBD	IBD was induced by feeding DSS	HRW, oral; Duration: 7 days	IL‐1β, IL‐12, TNF‐α↓	[174]
IBD	A rat model of IBD induced by colorectal administration of TNBS	HRS (0.2 mL/10 g), peritoneal injection/oral Lactulose (0.1, 0.15, 0.2 mL/10 g); Duration: Twice a day for 7 days	HMGB1/RAGE/NF‐κB↓, AMPK/mTOR and Nrf2/HO‐1 ↑	[175]
IBD	IBD was induced by feeding DSS	HRS (5 mL/kg BW) peritoneal injection; Duration: Twice a day for 7 days	ER stress↓, HO‐1↑	[90]
IBD	IBD was induced by feeding DSS	HRW, oral; Duration: 14 days	Total thiol, SOD, and CAT↑	[176]
IBD	IBD was induced by feeding DSS	Oral MgH_2_@EC@ES; Duration: 8 days	ATP↑	[177]
IBD	IBD was induced by feeding DSS	Oral SiH NPs (20 mg/kg); Duration: 8 days	ROS↓, Diversity of GM↑	[179]
UC	DSS‐induced chronic ulcerative colitis mice	HRW (0.8 ppm), oral; Duration: 63 days	TNF‐α and Harmful GM↓, GSH↑	[181]
IBD	IBD was induced by feeding DSS to the mice	HRS (3.0 ppm) 0.1 mL each intraperitoneal injection; Duration: 17 days	The epithelial expression of Nos2↓, intestinal‐SCFA‐producing bacteria↑, Production of SCFA↑	[182]
Intestinal I/R injury	A rat intestinal intussusception (II) model	HRS (5 mL/kg BW), intraperitoneally; Duration: single injection	TNF‐α, MDA↓, SOD↑	[188]
Intestinal I/R injury	The rat model of intestinal I/R injury	HRS (5 mL/kg BW), intraperitoneally; Duration: Two injections	MPO, MDA, IL‐6 and TNF‐α↓	[191]
Intestinal I/R injury	The rat model of intestinal I/R injury	HRGS (2 mL) injected by oral route, abdominal puncture, and the dorsal vein of the penis; Duration: single injection	MPO, MDA, IL‐6 and TNF‐α↓	[192]
Intestinal I/R injury	The rat model of intestinal I/R injury	Inhalation of 2% H_2_; Duration: 3 h	CCL2, IL‐1 β, IL‐6, and TNF‐α↓	[43]
Intestinal I/R injury	The rat model of intestinal I/R injury	HRS (5 mL/kg) intravenous injection; Duration: single injection	MPO, MDA, and NF‐κB↓	[195]
Intestinal I/R injury	The rat model of intestinal I/R injury	HRS intravenous injection (concentration: ≥0.6 mmol/L, ≥0.6 ppm) 10 or 20 mL/kg; Duration: single injection	NF‐κB/NLRP3 pathway↓	[120]
Intestinal I/R injury	The mice model of intestinal I/R injury	HRS (3 µmol/kg) intraperitoneal injection; Duration: one injection per day for 5 days	miR‐199a‐3p↓, IGF1/PI3K/Akt/mTOR↑	[199]
CRC	Stage IV CRC cancer patients	Inhalation of H_2_ (flow rate: 1.67 L/min; hydrogen purity: 99.99%); Duration: 3 h a day for 3 months	PD‑1^+^ CD8^+^↓, Mitochondria↑, PD‑1^−^ CD8^+^↑	[206]
CRC	CRC cell lines (ROK/SW480/HCT116) and xenograft mouse models	Inhalation of 66% H_2_ (66% H_2_ and 33% O_2_); Duration: 2 h a day for 21 days	AKT/SCD1↓	[213]
CRC	A colorectal cancer xenograft model	HRW, oral and oral gavage (200 µL daily); Duration: Lasts 14 days	MDA↓, CAT, and SOD↑	[214]

*Abbreviations*: CRC, colorectal cancer; ER, Endoplasmic reticulum; IBD, inflammatory bowel disease; IND, indomethacin; MgH_2_@EC@ES, a new core–shell structure of intestine‐targeted controlled hydrogen‐releasing MgH_2_ microcapsules by encapsulating MgH_2_ microparticles with ethyl cellulose, Eudragit S100 and Span‐83; SCFA, short‐chain fatty acids; TNBS, trinitrobenzene sulfonic acid; UC, ulcerative colitis.

#### 4.1.1 H_2_ Improves Intestinal Damage by Reducing ROS

**FIGURE 3:**
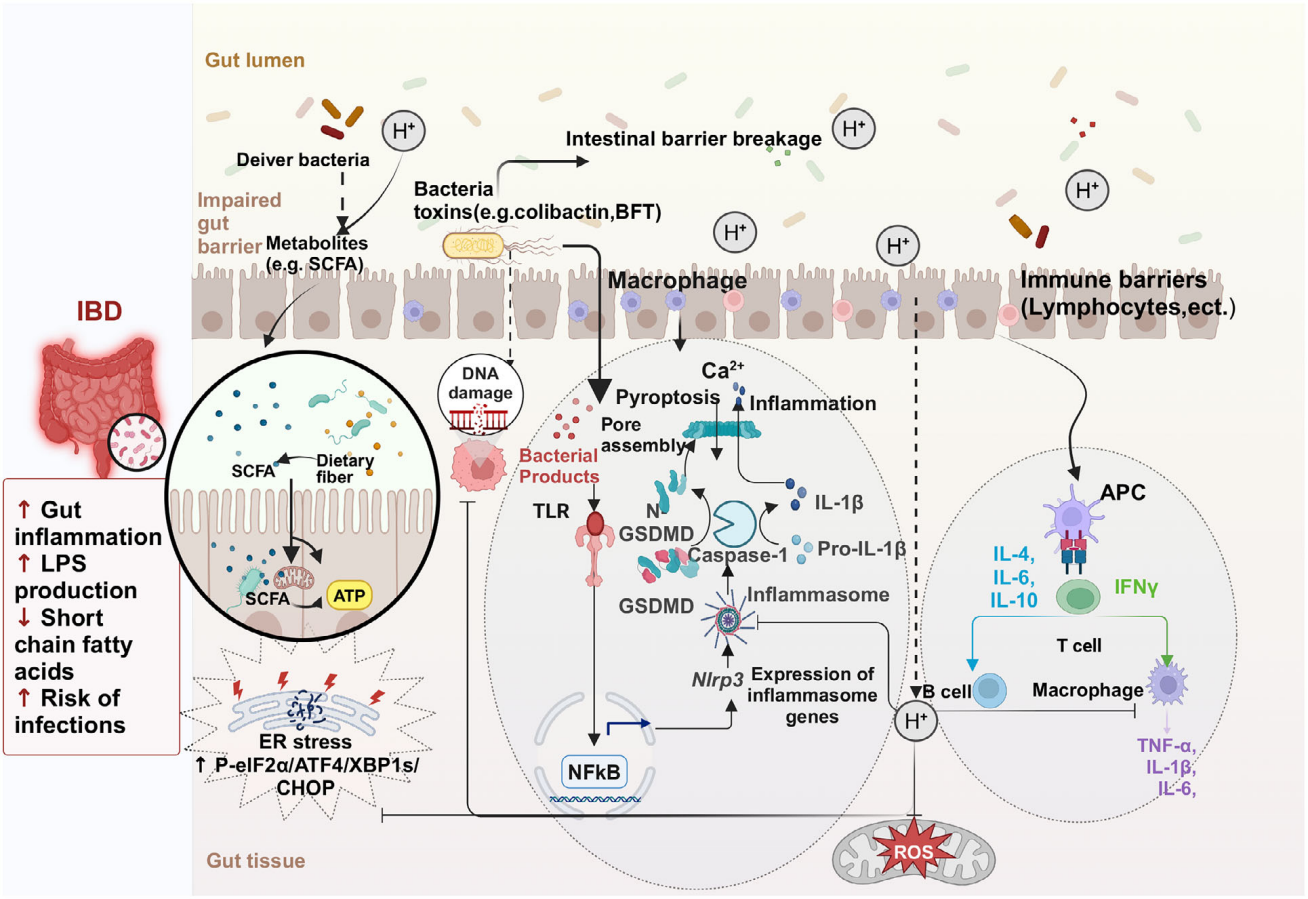
Mechanisms of molecular hydrogen alleviating IBD and intestinal injury. This illustration summarizes the process of hydrogen therapy for IBD and the key molecules and targets involved in the regulation process. Hydrogen promotes the metabolism of dietary fiber into SCFAs by GM, and the resulting SCFAs are absorbed into the colon via monocarboxylic acid transport proteins, ultimately providing energy to cells in the form of ATP. Bacterial toxins activate TLR expression which further activates and phosphorylates NF‐κB, promoting the release of inflammatory factors such as TNF‐α, IL‐6, IL‐1β, and so on, while NF‐κB translocates from cytoplasm to nucleus inducing transcriptional activation NLRP3 inflammatory vesicles leading to increased expression inflammatory factors as well as activation NLRP3 inflammasome producing reactive oxygen species (ROS). ROS overload is thought to be involved in IBD pathogenesis. Hydrogen not only directly inhibits ROS generation but also inhibits the NF‐kB signaling pathway, thus suppressing oxidative stress and inhibiting the release of proinflammatory factors. Moreover, hydrogen administration resulted in a significant decrease in the expression of eIF2 α, ATF4, XBP1s, and CHOP proteins, that is, hydrogen‐inhibiting endoplasmic reticulum stress. As there is an accumulation of M1‐like macrophages in the diseased area of IBD patients, which produce proinflammatory cytokines such as TNF‐α, IL‐6, IL‐1β, ROS, and so on, which perpetuate inflammatory damage to the intestinal mucosa, hydrogen administration reduces the level of TNF‐α, thus slowing down intestinal damage caused by IBD.

Nonsteroidal anti‐inflammatory drugs (NSAIDs) are widely utilized analgesics and are known to cause gastrointestinal damage, with a greater impact on the small intestine than the stomach. The prevention and treatment of NSAID enteropathy pose significant challenges, as there are currently no viable measures available to prevent initial damage caused by uncoupling of mitochondrial oxidative phosphorylation. Therefore, interventions should primarily focus on downstream inflammatory processes [166]. Considering the potential involvement of ROS in the pathogenesis of this condition, antioxidants hold promise for effectively preventing and treating NSAID‐induced enteropathy [167]. Akita et al. [168] discovered that oral HRW inhibited the production of cytokines, MPO, mucosal permeability, and ROS in the small intestinal mucosa induced by indomethacin, an NSAID drug. Additionally, HRW altered the metabolism of intestinal microorganisms and promoted the production of more favorable SCFA [168]. Jin et al. [169] found that HRW significantly activated the Nrf‐2 signaling pathway, suppressed ROS expression, attenuated NF‐κB signaling pathway activation, and exhibited a protective effect against chronic intestinal inflammation induced by lipopolysaccharide in rats (Figure 3). Hu et al. [170] showed that Electrolyzed Hydrogen Water (ERW) could control colonic inflammation and relieve gastroparesis by inhibiting overproduction of ROS and blocking the communication between oxidative stress and inflammation (Figure 3). Qiu et al. [171] showed that HRS treatment reduced intestinal damage and improved intestinal function in whole‐body radiation exposed mice by inhibiting oxidative stress‐induced injury and systemic inflammation, suppressing intracellular ROS production and blocking the mitochondrial apoptosis pathway (Figure 3). Chen et al. [172] demonstrated that HRW can reduce ROS production and alleviate intestinal oxidative stress by acting on the SIRT1/Nrf2/HO‐1 signalling pathway, thereby alleviating constipation. The above studies have demonstrated that despite the diverse delivery methods of H_2_, all of them effectively ameliorated intestinal damage by reducing ROS production (Table 3).

#### 4.1.2 H_2_ Improves Intestinal Damage by Inhibiting ER Stress

The ER stress occurring in the intestinal epithelial cells (IEC) contributes to the pathogenesis of IBD, and targeting ER stress represents a crucial approach to improving IBD [173]. According to Chen et al. [83], H_2_ reduced inflammation and organ damage in septic mice via reducing ER stress through the autophagic route. The DSS colitis model's speed, simplicity, repeatability, and controllability make it a popular choice in IBD research. Kajiya et al. [174] demonstrated that H_2_ significantly inhibited DSS‐mediated colonic tissue destruction with macrophage infiltration to prevent DSS‐induced colitis in mice. The AMPK/mTOR, HMGB1/RAGE/NF‐κB, and Nrf2/HO‐1 pathways have been elucidated as the molecular mechanisms involved in the dynamic balance between colonic injury [175]. Shen et al. [90] observed a significant reduction in the protein expression of p‐eIF2α, ATF4, XBP1s, and CHOP after intraperitoneal injection of HRS into a DSS‐induced IBD mouse model, indicating that HRS can inhibit ER stress (Figure 3). Furthermore, treatment with HRS resulted in a significant upregulation of HO‐1 expression, and the use of ZnPP reversed the protective effect of HRS [90] (Figure 3). LeBaron et al. [176] used DSS‐induced IBD mice as a model to investigate the therapeutic effects of the colitis drugs sulfasalazine and HRW alone and in combination, showing that sulfasalazine and HRW alone significantly decreased inflammation (high‐sensitive C‐reactive protein) and restored redox balance (total thiol, SOD, and CAT). There was a trend for the combination treatment to be more effective than either HRW or sulfasalazine alone. Furthermore, HRW tended to be as effective as, and often more effective than, sulfasalazine [176]. Liu et al. [177] developed for the first time a novel gut‐targeted controlled‐release H_2_ MgH_2_ microcapsule, MgH_2_@EC@ES, and evaluated the therapeutic efficacy of the microcapsule in a mouse model of ulcerative colitis, which showed that the microcapsule could dose dependently repair the IBD damage caused by DSS and repair the impaired mitochondrial energy metabolism function in colitis and that a high dose of MgH_2_@EC@ES was more effective than the first‐line drug 5‐aminosalicylic acid, however, the authors did not evaluate the effect of the combination of the two in the article [177] (Table 3).

#### 4.1.3 H_2_ Improves Intestinal Damage by Regulating GM and Increasing SCFA Production

Xue et al. [44] demonstrated that H_2_ altered the composition of the GM, leading to an increase in the relative abundance of *Mycobacterium anisopliae* and *Mycobacterium thickum*, while concurrently inhibiting the LPS/TLR 4/NF‐κB inflammatory pathway, thereby preventing HFD‐induced NAFLD. In a mice model of alcoholic liver disease, Liu et al. [160] found that H_2_ inhalation reduced hepatic oxidative stress, inflammation, and steatosis while also ameliorating liver injury. Moreover, H_2_ inhalation improved the GM by increasing the abundance of *Trichospiraceae Clostridium*, enhanced the integrity of the intestinal barrier, and mechanistically blocked the activation of the LPS/TLR 4/NF‐κB pathway in the liver [160]. Wang et al. [178] showed that H_2_ significantly altered the GM composition and ameliorated methamphetamine (METH)‐induced psychiatric disorders in METH abusers. These findings suggest that H_2_ is likely to have an ameliorative and therapeutic effect on disease by acting on GM.

Wei et al. [179] employed orally administered silicon H_2_ nanoparticles (SiH NPs) for targeted scavenging of ROS at inflammatory sites, thereby alleviating symptoms of IBD and restoring GM diversity by enhancing the abundance of beneficial bacteria. Takahashi et al. [180] found that treatment with H_2_ increased Lactinobactor and decreased Akkermansia, Gracilibacter, and Marvinbryantia, thereby ameliorating the effects of a HFD, which led to dysbiosis and small intestinal damage in senescence‐accelerated mice. Song et al. [181] discovered that giving HRW to mice with a chronic UC model created by DSS improved histopathological changes, raised Glutathione (GSH) concentration, and lowered TNF‐α levels. Additionally, HRW inhibited the overgrowth of pathogenic or conditionally pathogenic bacteria (*Enterococcus faecalis, Clostridium perfringens*, and *Bacteroides fragilis*) in DSS‐induced chronic UC. These results suggest that consuming HRW can modulate gut bacterial dysbiosis during the progression of chronic UC [181]. Meanwhile, Ge et al. [182] discovered that the administration of HRW activates the growth of SCFA‐producing bacteria and increases the production of SCFAs. This, in turn, activates the intracellular butyrate‐sensor peroxisome proliferator‐activated receptor γ signaling, leading to a reduction in epithelial expression of Nos2. Consequently, it promotes the restoration of an anaerobic environment in the colon and improves the DSS‐induced acute colitis model in mice [182]. Akita et al. [168] performed fecal microbiota transplantation using cecum contents obtained from HRW‐drinking mice and subsequently analyzed the SCFAs content in the cecum contents. The findings revealed that HRW exhibited not only a direct antioxidant effect but also an indirect anti‐inflammatory effect by augmenting the SCFAs content [168] (Figure 3).

Competitive interactions among natural GM are critical for maintaining colony stability. Probiotics or microbial transplants can modulate the intestinal microenvironment, facilitating the development of beneficial bacteria to reduce the proliferation of harmful bacteria, ultimately leading to the formation of a new intestinal immune homeostasis. Molecular H_2_ has demonstrated its impact on GM in both IBD and intestinal injury treatments, promoting the enrichment of beneficial bacteria and subsequently enhancing SCFA production to alleviate IBD and intestinal injury (Table 3).

### 4.2 Intestinal I/R Injury

H_2_ has been extensively investigated for its potential to mitigating I/R injury in various organs. For instance, Li et al. [94] demonstrated that HRS exerts a neuroprotective effect against whole brain I/R by upregulating Treg cells and downregulating the expression of miR‐21, miR‐210, and NF‐κB. Liu et al. [183] showed that HRS reduces apoptosis induced by skin I/R and improves flap survival modulation of the Bax/Bcl‐2 ratio and inhibition of the activated Apoptosis Signal‐Regulating Kinase 1/JNK pathway. Lu et al. [184] revealed that HRS attenuates hepatic I/R injury by inhibiting ER stress‐induced apoptosis. Zhang et al. [185] protected rats from liver I/R injury by activating the NF‐κB signaling pathway after 1 h of H_2_ (2%) inhalation before liver transplantation. Nie et al. [186] identify inhibition of oxidative stress and NLRP3‐mediated apoptosis as crucial mechanisms by which H_2_ attenuates myocardial I/R injury. Zhai et al. [187] demonstrated that oral administration of lactulose promotes bacterial H_2_ production in the gastrointestinal tract, leading to activation of Nrf2 expression and subsequent reduction of brain I/R in rats. These data suggest that both HRS and H_2_ may possess therapeutic potential for mitigating organ I/R injury through their anti‐inflammatory, antioxidant, apoptosis inhibition, and mediation of various signaling pathways.

The intestine is one of the organs most sensitive to I/R injury, which refers to the exacerbation of intestinal damage following the restoration of blood flow after ischemia [188]. Reduced contractile activity, increased microvascular permeability, and mucosal barrier failure are the hallmarks of intestinal I/R injury. These changes can ultimately lead to systemic inflammatory response syndrome, as well as multiorgan dysfunction and failure [189]. At present, limited research has been conducted on the therapeutic effect of H_2_ in treating intestinal I/R injury, and the underlying mechanisms are summarized below (Table 3).

#### 4.2.1 Through Inhibition of the NF‐κB/NLRP 3 Pathway, H_2_ Reduces Intestinal I/R‐Induced Coagulation Problems and Inflammation

**FIGURE 4:**
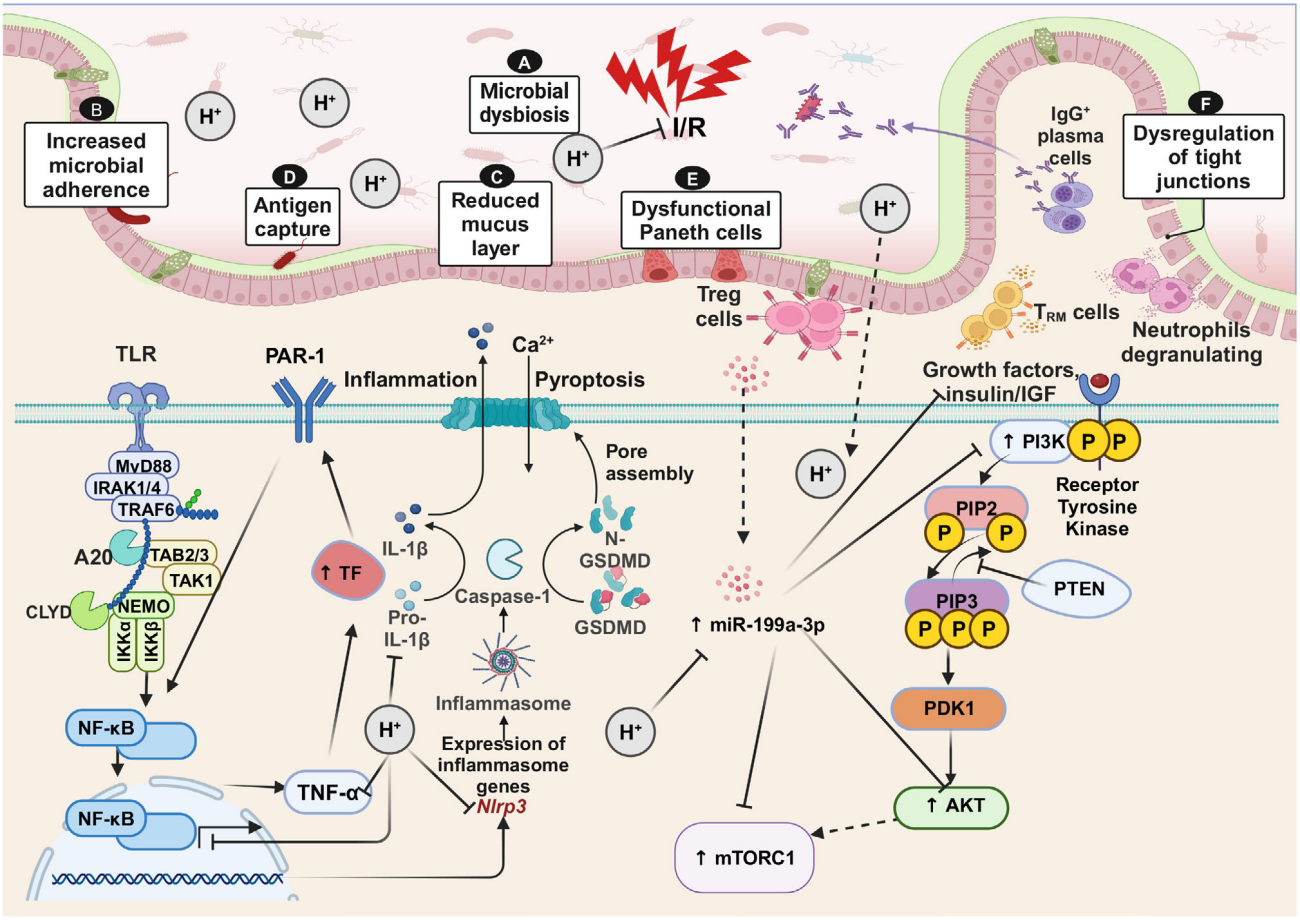
Mechanisms of molecular hydrogen reducing intestinal I/R injury. During intestinal I/R injury, bacteria, endotoxin, and reactive oxygen species enter the circulatory system and activate NF‐κB expression, leading to increased levels of TF, p‐NF‐κ B p65, NLRP3, Caspase‐1, and inflammatory factors. Hydrogen inhibits TNF‐α while elevating TF levels. This inhibition prevents the deleterious cycle of the inflammatory cascade and coagulation system activation by blocking the NF‐κB signaling pathway and its downstream molecule NLRP3 inflammatory vesicle expression. Furthermore, hydrogen reduces intestinal I/R damage by upregulating the expression of IGF‐1, PI3K, AKT, and mTOR proteins and downregulating the expression of miR‐199a‐3p. In summary, hydrogen ameliorates intestinal I/R injury by suppressing the NF‐κB/NLRP3 signaling pathway.

Organ and tissue damage are not the only things caused by intestinal I/R injury; it also throws off the balance of coagulation. Under physiological conditions, the coagulation system, anticoagulation system, and fibrinolytic system cooperate to maintain a dynamic balance [190]. MPO serves as an inflammatory biomarker, while TNF‐α and IL‐6 are classical proinflammatory factors during the inflammatory phase. Wu et al. [188] demonstrated that administration of HRW significantly reduces serum TNF‐α levels and mitigates intestinal mucosal injury in I/R rats (Figure 4). Eryilmaz et al. [191] observed that although the administration of HRS does not significantly alter the levels of MPO, MDA, IL‐6, and TNF‐α in intestinal I/R rats, it results in reduced tissue damage and apoptosis. In contrast, in another study by Shigeta et al. [192], intraluminal injection of H_2_‐rich glucose saline (HRGS) was found to be effective in inhibiting MDA production and thus attenuating intestinal I/R injury in rats. Protease‐activated receptor 1 on the surface of monocytes is activated by tissue factor (TF) expression upregulation, whereas TNF‐α initiates the cytokine cascade and encourages monocytes to express TF, creating a harmful cycle of inflammatory cascade and coagulation system activation [193]. One of the main mechanisms tying immunity and inflammation together is NF‐κB, which is particularly important for controlling proinflammatory proteins and taking part in the transcription of TF genes [194]. TNF‐α, IL‐6, and IL‐1 β are typical stimulatory signaling molecules of the NF‐κB pathway. Buchholz et al. [43] found that inhalation of 2% H_2_ attenuated I/R‐induced upregulation of the inflammatory mediators CCL2, IL‐1β, IL‐6, and TNF‐α during small intestinal transplantation in rats. Mao et al. [195] found that administration of HRS suppresses the expression of proinflammatory cytokines IL‐1β and TNF‐α, as well as inhibits the activation of NF‐κB in intestinal I/R mice, thereby attenuating the damage caused by intestinal I/R (Figure 4). Yang et al. [120] reported that in a rat model of intestinal I/R injury, elevated levels of TF, p‐NF‐κB p65, NLRP3, Caspase‐1, and inflammatory factors were observed. Intestinal I/R causes coagulation dysfunction and activates NF‐κB/NLRP3 signaling pathway in rats. However, treatment with HRS attenuates the inflammatory response and upregulates TF expression to improve coagulation disorders and alleviate intestinal injury induced by I/R [120] (Figure 4).

Oxidative stress plays an important role in intestinal I/R injury and HRS has been shown to protect the intestine from I/R injury by inhibiting lipid oxidation, mitigating intestinal inflammation, reducing oxidative stress, and inhibiting apoptosis [43, 188, 196]. Further research is necessary to determine the exact mechanism and signaling pathway that underlie H_2_’s protective effects in intestinal I/R injury (Table 3).

#### 4.2.2 H_2_ Downregulates the Expression of miR‐199a‐3p to Activate IGF‐1/PI3K/mTOR Pathway and Reduce Intestinal I/R Injury

MicroRNAs regulate 30–80% of the human genome and serve as crucial biomarkers in the pathogenesis of human diseases, including intestinal I/R. In rats suffering from myocardial I/R injury (MI/RI), Zhang et al. [197] demonstrated that miR‐98‐5p targets TLR4, which inhibits TLR4 and activates the PI3K/Akt signaling pathway, protecting MI/RI. Yao et al. discovered a significant upregulation of miR‐199a‐3p following intestinal I/R injury, which was found to target insulin‐like growth factor‐1 (IGF‐1), mTOR, and PI3K regulatory subunit 1 (PIK3r1) that encodes a key PI3K part of the gene. In other words, miR‐199a‐3p targets the IGF‐1/PI3K/Akt/mTOR pathway involved in cell growth and division [198, 199]. The protein expression levels of these targeted molecules were generally reduced after intestinal I/R injury as evidenced by decreased expression of IGF‐1, PI3K, Akt, and mTOR. This suggests that miR‐199a‐3p may inhibit the prosurvival pathway. However, HRS pretreatment (Before intestinal I/R surgery in mice, intraperitoneal injection of HRS (3 µmol/kg) was administered for 5 days in a row.) significantly increased the protein levels of these four members and exhibited a protective effect similar to that observed with miR‐199a‐3p inhibitor treatment. In conclusion, HRS treatment may protect IECs from I/R injury by downregulating the expression of miR‐199a‐3p and activating the IGF‐1/PIK3r1/mTOR‐related cell survival pathway [199] (Figure 4 and Table 3).

### 4.3 Colorectal Cancer

Cancer is a significant global public health issue, and CRC ranks as the second leading cause of cancer‐related mortality [200]. Current therapeutic approaches for CRC include surgery, radiotherapy, chemotherapy, and targeted therapy [201]. While these anticancer treatments have demonstrated efficacy in enhancing the prognosis of patients with CRC, a significant number of patients encounter unfavorable side effects following surgery and chemotherapy. Growth factors, PI3K/AKT/mTOR, and MAPK are examples of signaling pathways that are crucial to the pathophysiology of CRC [202]. Research has indicated that H_2_ inhibits tumor cell activity, proliferation, invasion, and migration through various molecular mechanisms, in a manner that depends on both dose and time. Additionally, it has been shown to mitigate nephrotoxicity in patients undergoing radiotherapy and chemotherapy [203]. According to Wang et al. [76], H_2_ dramatically slows tumor growth in vivo and, in a concentration‐dependent manner, decreases cell proliferation and induces apoptosis in vitro. Furthermore, H_2_ lowers lung tissue levels of ROS, TNF‐α, IL‐1 β, IL‐8, and IL‐13 and elevates the SOD expression. Further evidence of the suppression of SMC3 expression in A549 and H1975 cells was provided by immunohistochemical labeling [76]. Yang et al. [9] demonstrated that administration of HRW in a xenograft mouse model results in a significant reduction in both the volume and weight of endometrial tumors, thereby inhibiting the growth of endometrial cancer through ROS/NLRP 3/caspase‐1/GSDMD‐mediated apoptosis. As a result, H_2_ therapy has drawn more attention and is now acknowledged as a potentially effective cancer treatment strategy.

#### 4.3.1 H_2_ Improves Prognosis by Restoring Depleted CD8^+^ T Cells in Patients with CRC Cancer

**FIGURE 5:**
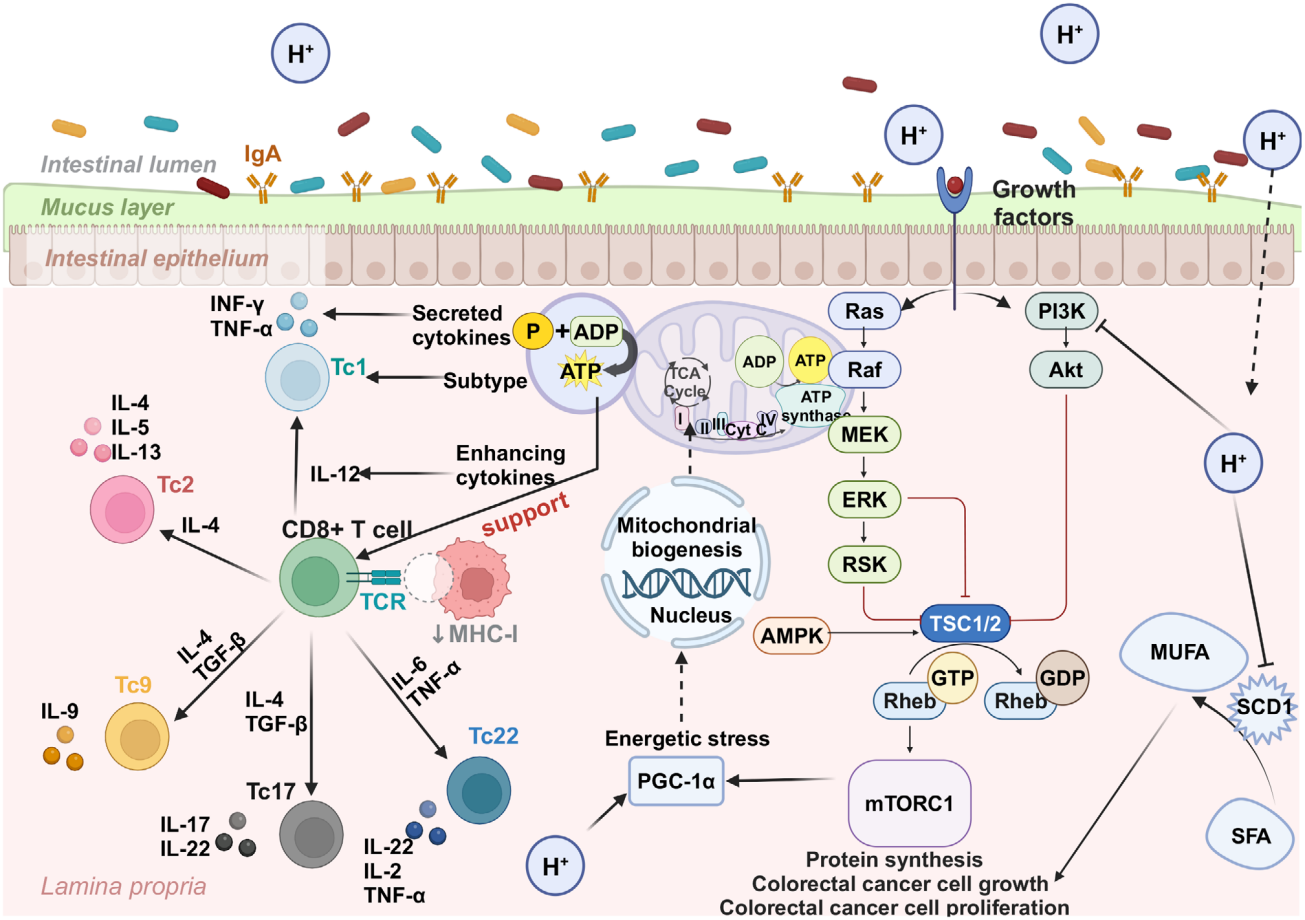
Mechanisms of molecular hydrogen inhibiting CRC growth. CD8+ T cells assist the host in resisting viral infections and tumor cell invasion. However, prolonged exposure to persistent antigens or chronic inflammation leads to a loss of function in cytotoxic T lymphocytes (CTL) and an identity crisis in memory T cells (TM), resulting in terminal exhausted T state. Hydrogen directly counteracts mitochondrial dysfunction caused by PGC‐1α inactivation, which provides ATP to terminal exhausted T after mitochondrial function is restored, and increases the amount of terminal exhausted T to improve the prognosis of colorectal cancer patients. The PI3K/AKT/mTOR signaling pathway plays a crucial role in tumourigenesis and development, with overactivation of PI3K promoting rapid growth and reproduction of cancer cells. mTORC1 regulates the balance between cellular anabolic and catabolic metabolism, which is associated with cell growth. Hydrogen can directly inhibit the expression of PI3K and PAKT, thereby suppressing downstream signaling molecule mTORC1 expression to inhibit colorectal cancer cell growth. Meanwhile, hydrogen reduces cancer stem cell (CSCs) activity in CRC by targeting inhibition of the p‐AKT/SCD1 pathway.

High infiltration of CD8^+^T cells in CRC indicates a favorable prognosis and positive response to immunotherapy [204]. However, prolonged interactions between T cells and tumors impair T‐cell competence, while PGC‐1α inactivation leads to mitochondrial dysfunction, thereby compromising the immune activity of CD8^+^ T cells. It has been shown that H_2_ can activate PGC‐1α to restore mitochondrial function and rescue depleted CD8^+^T cells [205]. Programmed cell death 1 (PD‐1) is considered a marker of T‐cell depletion and is associated with poor prognosis in various cancers. In a clinical study conducted by Akagi and Baba [206], involving 55 patients diagnosed with stage IV CRC, it was observed that inhalation of H_2_ for 3 h/day and concurrent chemotherapy in the hospital resulted in a reduction in the abundance of exhausted terminal PD‐1^+^CD8^+^ T cells. This depletion of CD8^+^ T cells may be attributed to the activation of mitochondria through PGC‐1α, leading to an increase in the population of active terminal PD‐1^−^CD8^+^ T cells and ultimately improving patient prognosis [206] (Figure 5 and Table 3).

#### 4.3.2 H_2_ Inhibits CRC Cell Proliferation by Suppressing the AKT/SCD1 Pathway

Stearoyl‐CoA desaturase 1 (SCD 1) serves as the rate‐limiting enzyme in the synthesis of monounsaturated fatty acids and oleic acid from saturated fatty acids and stearic acid [207]. All malignancies share the trait of cell proliferation, which is dependent on fatty acids for the creation of signaling molecules and cell membranes [208]. Given its high expression levels in various cancer types and pivotal role in regulating fatty acid metabolism to facilitate cancer cell growth, SCD 1 is regarded as a promising therapeutic target for cancer treatment [209]. In addition, SCD1 has been identified as a marker for CSCs in CRC [210], and the SCD 1 inhibitors effectively attenuate the stemness properties of CSCs [211]. Protein kinase B, also referred to as Akt, is a 57‐kDa serine/threonine kinase that functions as a signaling molecule for cell growth and development. By transferring signals from upstream regulatory proteins like PI3K to several downstream effectors like glycogen synthase kinase 3 β (GSK3 β), which in turn interacts with other compensatory signaling pathways, the Akt pathway functions as an effective mediator [212]. The PI3K/AKT pathway controls a variety of cellular processes, including autophagy, apoptosis, and proliferation. The prevalence of human diseases resulting from dysregulated Akt signaling has prompted the active development of small molecule inhibitors targeting PI3K and Akt.

The phosphorylation of AKT and the elevation of pAKT levels are commonly observed in most malignancies. In their study, Zhang et al. [213] investigated the impact of H_2_ on SCD1 and PI 3K/AKT signaling pathways in CRC cell lines (RKO, SW480, and HCT116), revealing a significant downregulation of SCD1 in H_2_‐treated CRC cells. Interestingly, H_2_ exhibits distinct effects on different CRC cells; it enhances PI3K expression in RKO cells while diminishing it in SW480 and HCT116 cells. Moreover, H_2_ decreases the expression of both PI3K and p‐AKT specifically in SW480 and HCT116 cells. Therefore, H_2_ demonstrates its potential to inhibit the PI3K/Akt pathway for reducing proliferation in CRC cells, and this reduction in AKT phosphorylation may induce inhibition of downstream signaling [213]. AKT activator SC79 was used by Zhang et al. [213] to treat RKO, SW480, and HCT116 cells. This led to a dose‐dependent upregulation of pAKT expression. Treatment of all CRC cells with SC79 reverses the inhibitory effect of H_2_ on cell proliferation and significantly upregulated SCD1 and pAKT expression in a dose‐dependent manner. These findings suggest that H_2_ inhibits CRC cell survival by reducing pAKT levels and targets the AKT/SCD1 pathway to inhibit tumor growth both in vitro and in vivo [213] (Figure 5). Asgharzadeh et al. [214] treated a CRC mouse model with H_2_ water and 5‐fluorouracil individually, while also setting up a combination treatment group to explore the effects. The results showed that H_2_ water alone significantly improved detected antioxidant markers (SOD and CAT) and reduced MDA levels. However, the combination of H_2_ water and 5‐fluorouracil significantly attenuated MDA levels more effectively than 5‐fluorouracil alone [214]. This also highlights the potential of H_2_ therapy as an adjunct to cancer treatment (Table 3).

## 5 Preventive and Therapeutic Applications for Other Diseases

Molecular H_2_ assumes a critical function not only in the prevention and management of gastrointestinal disorders but also in demonstrating therapeutic advantages across a multitude of physiological systems within the human organism. This highlights the multifaceted therapeutic attributes of H_2_ and its prospective role as a notable medical intervention. Comprehensive investigations have clarified the mechanisms that underlie the therapeutic effects of H_2_, revealing its capacity to neutralize deleterious free radicals, modulate intracellular signaling pathways, and promote cellular viability. Recent research indicates that the therapeutic ramifications of H_2_ have been thoroughly examined across an array of pathological conditions, encompassing respiratory ailments, disorders of the central nervous system, cardiovascular abnormalities, hepatic and renal pathologies, as well as cancer‐related diseases.

### 5.1 Lung Diseases

Lung diseases represent a diverse group of conditions that impact the respiratory system, characterized by distinct etiologies, clinical manifestations, and treatment strategies. These conditions often involve intricate interactions between environmental factors and comorbidities, presenting substantial challenges for accurate diagnosis and effective management. In recent years, H_2_ has gained attention as a promising therapeutic intervention, exhibiting significant potential in ameliorating various lung diseases and providing novel insights into their management. Lung injuries comprise a wide array of pathologies resulting from a multitude of etiological factors, including but not limited to mechanical trauma, chemical exposure, and lifestyle‐related influences. A comprehensive comprehension of these pulmonary injuries is imperative for accurate diagnosis and effective therapeutic interventions. Studies have shown that H_2_ possesses notable therapeutic potential in mitigating various types of lung injuries. In a hyperoxia‐induced lung injury model, mice exposed to a high‐oxygen (98%) environment were treated with 2% H_2_ gas inhalation, which activated the Nrf2 pathway and protected the lungs from oxidative stress‐induced damage [215]. Similarly, Nrf2 is a key mediator in the therapeutic effects of H_2_ gas on sepsis‐induced lung injury. This was demonstrated by Yang et al. [216], who compared the responses of wild‐type and Nrf2‐deficient mice. Inhalation of 2% H_2_ gas for 1 h significantly elevated the levels of SOD, CAT, and HO‐1 while reducing HMGB1 levels in wild‐type mice. It is noteworthy that these therapeutic effects were not observed in Nrf2‐deficient mice, thereby emphasizing the essential role of Nrf2 in mediating the therapeutic properties of H_2_ gas [216]. The therapeutic effectiveness of H_2_ gas in treating sepsis‐induced lung damage in mice has been thoroughly demonstrated; nevertheless, its preventive capabilities require additional experimental investigation. Wang et al. [217] proposed an innovative approach by integrating H_2_ gas with engineered microalgae, providing a promising avenue for the prevention of sepsis‐associated lung injury.

ALI is a severe and life‐threatening clinical condition. Numerous studies have confirmed that H_2_ alleviates lung injury through various mechanisms. Nrf2 serves as a critical target in H_2_ gas therapy for both hyperoxia‐induced lung injury and sepsis‐related lung injury. Similarly, in a seawater‐induced ALI model, Nrf2 also plays a vital role. Inhalation of 2% H_2_ gas for 2 h significantly improved partial pressure of oxygen, reduced inflammatory cytokines such as TNF‐α, IL‐1β, and IL‐6, as well as the activities of MDA and MPO, while upregulating the expression of Nrf2 and HO‐1 [46]. Li et al. [218] performed an extensive study on the impact of H_2_ gas on LPS‐induced ALI and found that H_2_ gas significantly improved survival rates, enhanced lung function, reduced weight loss, and diminished the levels of proinflammatory cytokines such as IL‐6, TNF‐α, and IL‐1β in mice. Furthermore, H_2_ gas offered protection against lung damage by influencing AMPK activity and decreasing the expression of Drp1 and Caspase‐3 [218]. Yin et al. [219] conducted both in vivo experiments using an LPS‐induced ALI mouse model and in vitro experiments with RAW264.7 macrophages. The results showed that H_2_ gas inhalation increased the 72‐h survival rate of mice to 80%, reduced lung injury, and suppressed the release of inflammatory cytokines. The in vitro experiments further demonstrated that H_2_ gas inhibited the expression of ROS, NO, TNF‐α, IL‐6, and IL‐1β in macrophages, while also reducing TLR4 expression and NF‐κB activation [219]. Fu et al. [113], employing an LPS‐induced ALI rat model and human pulmonary microvascular endothelial cells, demonstrated that HRS mitigates ALI by downregulating the mTOR/TFEB signaling pathway.

Chronic obstructive pulmonary disease (COPD), the third leading cause of mortality globally, is marked by a high prevalence and significant healthcare burden. Smoking and exposure to toxic particulates represent the primary risk factors. Current therapeutic strategies encompass smoking cessation, inhalation therapy, and pulmonary rehabilitation [220]. Nonetheless, H_2_ gas has emerged as a promising therapeutic option for the management of COPD. Animal studies suggest that H_2_ gas inhalation may serve as an effective strategy for managing COPD. In a mouse model of cigarette smoke solution‐induced COPD‐like injury, inhalation of 42% H_2_ gas for 75 min twice daily over 9 weeks resulted in a 100% survival rate, compared with 80% in the untreated group. Histopathological evaluations indicated that H_2_ gas treatment reduced emphysema‐like changes and pulmonary inflammation [221]. Su et al. [222] developed a COPD rat model using cigarette smoke and LPS, observing that a 2‐h daily inhalation of H_2_ gas significantly enhanced lung function and decreased inflammatory cytokine levels. The therapeutic effects were attributed to the suppression of M1 macrophage polarization and the promotion of M2 macrophage polarization, effectively mitigating pulmonary inflammation. In addition, in a COPD mouse model, inhalation of a gas mixture containing 42% H_2_, 21% oxygen, and 37% nitrogen significantly reduced emphysema, collagen deposition, and goblet cell hyperplasia, while inhibiting the activation of ERK1/2 and NF‐κB signaling pathways [222].

Molecular H_2_ has shown multiple therapeutic advantages in treating pulmonary diseases, including its ability to directly repair lung tissue damage and provide comprehensive protective effects by regulating molecular signaling pathways. Its ability to modulate oxidative stress and inflammatory responses in ALI, along with its role in promoting tissue repair and maintaining immune balance in COPD, underscores its potential to influence disease progression at the level of pathological mechanisms.

### 5.2 Central Nervous System Diseases

Molecular H_2_ has been extensively studied for its potential neuroprotective effects in various neurological disorders, with its therapeutic efficacy primarily attributed to its strong antioxidant, anti‐inflammatory, and antiapoptotic properties. These mechanisms help slow the progression of neural damage. H_2_ gas has shown significant benefits in neurodegenerative diseases, acute neurological conditions, cognitive impairments, mood disorders, and neuropathic pain. Oxidative stress plays a pivotal role in the pathogenesis of neurodegenerative diseases. It not only triggers lipid peroxidation and DNA damage but also promotes neuroinflammation, further aggravating neuronal injury—key features of various neurodegenerative diseases. Molecular H_2_ mitigates neuronal damage through its anti‐inflammatory and antioxidant properties, while also regulating cellular signaling pathways to enhance neuronal integrity. Due to its high biosafety, H_2_ is regarded as a promising strategy for slowing the progression of neurodegenerative diseases and serving as a long‐term therapeutic approach [223]. For example, Hou et al. [104] studied the effects of HRS on cognitive impairment in APP/PS1 mice and found that, although HRW could not clear Aβ, it significantly improved the cognitive abilities of the mice, with this improvement exhibiting gender dependence. In contrast, Zhang et al. [224], using PdH nanoparticles in 3×Tg‐AD mice, reported that H_2_ gas significantly inhibited the overexpression of APP, BACE1, and sAP, thereby reducing Aβ production. This intervention effectively halted the progression of AD, alleviating cognitive impairment, synaptic deficits, and neuronal death [224]. Moreover, Lin et al. [225] conducted a 7‐month treatment with HRW in 3×Tg‐AD mice and demonstrated that it prevented synaptic loss and neuronal death, reduced amyloid plaques, hyperphosphorylated tau protein, and neurofibrillary tangles. Additionally, HRW improved disturbances in brain energy metabolism and dysbiosis of GM while mitigating inflammatory responses [225]. The interaction between the gut and brain axis is believed to play a critical role in alleviating neuroinflammation, with the regulation of GM by HRW considered a key mechanism underlying its neuroprotective effects.

In the treatment of acute neurological disorders, rapid diagnosis, and timely intervention are essential. Studies have demonstrated that H_2_ therapy provides significant neuroprotective effects in models of acute neurological conditions, including cerebral I/R injury, subarachnoid hemorrhage (SAH), and traumatic brain injury (TBI). For instance, in a cerebral I/R injury model, Lee et al. [226] reported that drinking HRW reduced Bax and TNF‐α levels in the hippocampus of mice, decreased ROS levels, and improved anxiety and memory performance. Moreover, intraperitoneal injection of HRS showed notable efficacy by promoting HO‐1 expression, increasing SOD levels, and reducing MDA levels, thereby mitigating oxidative stress‐induced brain damage [227]. In an SAH model, exposure of mice to a 3% H_2_ environment activated Nrf2, upregulated GpX4 expression, and inhibited TLR4 activation, significantly alleviating neurological damage [228]. Similarly, intraperitoneal injection of HRS effectively suppressed the NF‐κB pathway and the formation of the NLRP3 inflammasome [50], highlighting the multitarget regulatory effects of H_2_ in providing neuroprotection against SAH. Moreover, Wang et al. [229] demonstrated using a microvascular endothelial cell model that H_2_ gas activates the PI3K/AKT/GSK3β signaling pathway, thereby enhancing cell survival and providing new insights into the treatment of TBI. In the context of neuropathic pain, molecular H_2_ also shown promising therapeutic effects. A 2022 study reported that HRW alleviated inflammatory pain in mice and simultaneously improved associated depressive and anxiety‐related behaviors [230]. Additionally, Lian et al. [231] found that in mice with chemotherapy‐induced neuropathic pain caused by oxaliplatin, drinking HRW significantly reduced inflammation by inhibiting the LPS–TLR4 pathway and decreasing the expression of TNF‐α and IL‐6. Furthermore, HRW consumption improved GM composition by increasing the abundance of beneficial bacteria such as *Lachnospiraceae*, *Lactobacillaceae*, and *Ruminococcaceae*, while promoting the production of SCFA. These changes alleviated neuropathic pain through the gut–brain axis [231]. Overall, H_2_, by regulating immune responses, protecting neurons, and modulating the gut–brain axis, exhibits multifaceted therapeutic effects and holds significant promise as a potential treatment for central nervous system diseases.

### 5.3 Cardiovascular System Diseases

Cardiovascular diseases have always been a major factor contributing to global morbidity and mortality, necessitating a comprehensive management strategy that includes drug therapy, lifestyle changes, and innovative treatment approaches. However, existing traditional treatment strategies urgently need innovative breakthroughs. Molecular H_2_, as a novel bioactive molecule, has significant therapeutic potential for various cardiovascular conditions, including hypertension, atherosclerosis, myocardial I/R injury, and myocardial infarction.

Hypertension, as a chronic condition, is a critical risk factor for the development of various cardiovascular diseases and, if left uncontrolled, can lead to severe health complications. A study demonstrated that intermittent hypoxia rats inhaling 3% H_2_ gas for 2 h daily over 35 days showed significantly reduced levels of 8‐hydroxy‐2‐deoxyguanosine and increased SOD levels. These findings indicate that H_2_ gas alleviates oxidative stress, improves vascular dysfunction, and effectively lowers blood pressure without noticeable side effects [232]. Furthermore, in a rat model of hypertension induced by nephrectomy, inhalation of 1.3% H_2_ gas successfully controlled blood pressure by reducing sympathetic nervous system activity and enhancing parasympathetic nervous system activity [233]. Atherosclerosis is a disease characterized by persistent inflammation of the arterial walls, with its progression being heavily influenced by inflammatory responses. To overcome the limitations of current therapeutic approaches, researchers have developed nanomaterial‐assisted H_2_ delivery systems, which have significantly enhanced antioxidant and anti‐inflammatory efficacy. In ApoE−/− mice models, intraperitoneal injection of HRS was shown to inhibit the expression of Lectin‐like Oxidized Low‐Density Lipoprotein Receptor 1 (LOX‐1) and the activation of NF‐κB, effectively preventing the onset of atherosclerosis [234]. While existing medications can alleviate inflammation and reduce lesions, their limited efficacy and stability hinder broader application. To address these challenges, Xu and colleagues [235] developed a Palladium Hydride‐Tellurium (PDH‐Te) nanozyme that not only exhibits potent ROS‐scavenging abilities but also continuously releases H_2_ gas to suppress proinflammatory cytokine release, thereby significantly improving atherosclerosis. Additionally, H_2_ has been shown to regulate hypercholesterolemia, a key risk factor for atherosclerosis. For instance, intravenous injection of H_2_ nanobubble solution slowed disease progression with good safety and no adverse effects on animal survival rates [236].

In a myocardial ischemia model, intraperitoneal injection of 10 mL/kg HRS prior to reperfusion significantly reduced oxidative stress by modulating the total oxidative status and total antioxidant status, thereby minimizing myocardial cell damage and degeneration [237]. Another study showed that perfusion of H_2_‐rich Krebs‐Ringer solution (H_2_ concentration: 0.6 mmol/L, pH: 7.3) in rat hearts activated the JAK2–STAT3 signaling pathway while downregulating the p‐STAT1/STAT1 pathway, thus reducing myocardial apoptosis [238]. Additionally, HRS mitigated I/R injury by promoting mitochondrial autophagy via the PINK1/Parkin pathway to eliminate damaged mitochondria [239]. Nie et al. [151] developed H2‐PFOB NEs with a high H_2_‐loading capacity that deeply penetrated ischemic myocardium, significantly enhancing antioxidant and anti‐inflammatory effects and demonstrating superior therapeutic efficacy. When combined with low‐intensity focused ultrasound‐induced H_2_ release, the therapeutic effects of H2‐PFOB NEs were further enhanced [151].

In cardiac arrest (CA) and cardiopulmonary resuscitation models, H_2_ gas has also demonstrated protective effects on cardiac tissue by reducing injury markers and inflammation. Gong et al. [240] reported that inhalation of 2% H_2_ gas significantly reduced levels of myocardial injury markers, such as creatine kinase‐MB and cardiac troponin‐T, while protecting myocardial tissue from further damage by inhibiting autophagy. Furthermore, in a myocardial infarction model, daily inhalation of 2% H_2_ gas for 3 h over 28 days effectively suppressed the activation of the NLRP3 inflammasome, reduced cardiac fibrosis, and improved cardiac function [241]. These studies suggest that H_2_ therapy may serve as a viable adjunct to conventional treatments, offering new avenues for myocardial protection and recovery. The integration of nanomaterial‐assisted H_2_ delivery technologies has demonstrated significant potential to enhance therapeutic efficacy and offers promising prospects for achieving precision treatment of cardiovascular diseases.

### 5.4 Liver and Kidney Diseases

The liver and kidneys, as vital organs responsible for metabolism and detoxification, are crucial for maintaining overall health. Liver and kidney diseases are often interrelated, frequently cooccurring due to shared pathophysiological mechanisms and common risk factors. Therefore, preserving the health of these organs and identifying associated risk factors at an early stage are essential for safeguarding overall well‐being. NAFLD not only affects the liver but may also have systemic impacts, including on the kidneys and other organs. Studies have demonstrated that administering 0.6 mmol of HRS to NAFLD rats improves insulin sensitivity, enhances glucose tolerance, reduces liver enzyme and lipid levels, and achieves therapeutic effects by inhibiting TNF‐α and IL‐1β in the liver while upregulating PPARα and PPARγ expression [242]. Furthermore, Liu et al. [243] found that the therapeutic effects of H_2_ gas on NAFLD are dose dependent, with 4% H_2_ outperforming 67% H_2_ in reducing liver enzyme levels Alanine Aminotransferase (ALT) and Aspartate Aminotransferase (AST) and lipid accumulation. In another study, Xue et al. [44] demonstrated that inhalation of 4% H_2_ in an NAFLD rat model significantly lowered plasma LPS levels, inhibited the LPS/TLR4/NF‐κB signaling pathway to reduce liver inflammation, and enhanced intestinal barrier function by upregulating Zo‐1 and occludin expression. It also positively regulated the GM, increasing the Bacteroidetes/Firmicutes ratio, thereby highlighting the crucial role of the gut–liver axis in H_2_’s therapeutic effects on NAFLD [44]. H_2_ has also shown potential in mitigating chemotherapy‐induced liver injury. For instance, Yang et al. [244] observed that CRC patients receiving mFOLFOX6 chemotherapy exhibited stable liver function tests (ALT, AST, Alkaline Phosphatase (ALP)) after drinking HRW, indicating its hepatoprotective effects. Similarly, Gao et al. [245] demonstrated that injecting HRS in rats effectively reduced ALT and AST levels caused by doxorubicin, decreased ROS and MDA production, and regulated the Bax/Bcl‐2 ratio to alleviate inflammation and apoptosis. Li et al. [246] also proposed the potential protective effects of H_2_ against cisplatin‐induced side effects, although further research is needed to validate this hypothesis.

Chronic kidney disease (CKD) is a progressive disease whose major contributing factors include diabetes and hypertension. Studies suggest that H_2_ not only holds therapeutic potential for managing diabetes and hypertension but also effectively aids in the prevention and treatment of CKD. For instance, in CKD rats, drinking HRW significantly reduced plasma Monocyte Chemoattractant Protein‐1 (MCP‐1) levels [247]. In a mouse model of kidney injury induced by a high‐oxalate diet, HRW consumption markedly improved serum creatinine, blood urea nitrogen, and kidney injury markers such as kidney injury molecule‐1 (KIM‐1) and neutrophil gelatinase‐associated lipocalin (NGAL), while reducing proinflammatory cytokines and oxidative stress markers. HRW has been shown to mitigate renal injury by inhibiting key signaling pathways, including PI3K/AKT, NF‐κB, and TGF‐β [48]. Meanwhile, H_2_ has also demonstrated potential in the prevention of CKD [248]. In glycerol‐induced acute kidney injury models, inhalation of both 4 and 67% H_2_ gas demonstrated enhanced antioxidant, anti‐inflammatory, and antiapoptotic effects. Although no clear dose‐response relationship was observed, higher concentrations of H_2_ gas (67%) produced more pronounced improvements in kidney histology and morphology compared with lower concentrations (4%) [249]. Similarly, in I/R injury models, HRS treatment significantly reduced renal fibrosis and preserved kidney function [250]. Another study comparing short‐term and long‐term HRS injections revealed that intraperitoneal HRS increased the Bcl‐2/Bax ratio and inhibited the expression of apoptotic proteins such as caspase‐3, caspase‐8, and caspase‐9, but long‐term HRS treatment was more effective in promoting renal recovery than short‐term treatment [251], as it sustained the suppression of inflammation and apoptosis. These findings underscore the dose‐dependent nature of H_2_ therapy in liver and kidney diseases. Moreover, prolonged treatment may further enhance therapeutic outcomes, providing theoretical support for the application of H_2_ gas in metabolic diseases.

### 5.5 Cancer

Cancer encompasses a group of complex diseases characterized by uncontrolled cell growth and proliferation, with diverse manifestations and underlying mechanisms. While traditional approaches such as surgery, chemotherapy, and radiotherapy remain the cornerstone of cancer treatment—particularly effective for early‐stage cancers—their limitations have prompted researchers to explore innovative therapies. As a result, there has been a growing interest in developing novel adjunctive therapies with high safety profiles and minimal side effects. Advances in targeted therapy, immunotherapy, gene therapy, nanomedicine, and stem cell therapy have opened new avenues for cancer treatment. However, managing side effects continues to pose a significant challenge. Molecular H_2_, with its excellent safety profile and broad therapeutic potential, presents a promising complementary approach to cancer treatment. Preliminary progress has already been achieved in its application to various types of cancer.

As noted earlier, the therapeutic effects of H_2_ were initially identified through hyperbaric H_2_ therapy for treating skin cancer [2]. Since then, its therapeutic efficacy has been validated in various cancers, including CRC, lung cancer, gastric cancer, and breast cancer. For example, Zhang et al. [252] reported that H_2_ gas increased apoptosis in A549 cells while reducing the expression of XIAP and BIRC3 proteins in studies on A549 cells and their nude mouse models. Furthermore, inhalation of 60% H_2_ gas significantly reduced tumor volume in experimental mice [252]. Meng et al. [77] extended these findings by investigating the effects of H_2_ gas on A549 and H1975 lung cancer cells. They discovered that H_2_ gas significantly enhanced apoptosis by inhibiting CD47 expression and suppressed cell growth, invasion, and migration [77]. These findings further reinforce the potential role of H_2_ therapy in lung cancer treatment, particularly in modulating apoptosis‐related pathways.

In gastric cancer research, Zhu et al. [10] found that H_2_ gas downregulated the expression of lncRNA MALAT1 and EZH2 while upregulating miR‐124‐3p in Human Gastric Cancer Cell Line MGC‐803 (MGC‐803), Human Gastric Cancer Cell Line BGC‐823 (BGC‐823) gastric cancer cell lines and gastric cancer mouse models, thereby significantly inhibiting tumor growth and reducing cancer cell proliferation and migration. The potential of combination therapies has also been validated. For instance, combining platinum nanocolloid (Pt‐nc) with H_2_ gas effectively inhibited the growth of human promyelocytic leukemia HL60 cells and NUGC‐4 cells derived from human gastric adenocarcinoma [253]. Chu et al. [79] demonstrated that H_2_ gas exerts selective effects on HeLa cervical cancer cells and HaCaT keratinocytes. In a HeLa xenograft mouse model, continuous H_2_ gas inhalation significantly reduced tumor growth. Cell‐based experiments revealed that H_2_ gas selectively increased apoptosis in HeLa tumor cells while having minimal impact on nontumor cells such as HaCaT keratinocytes [79]. This selective action highlights the potential of H_2_ gas as a safer option for cancer therapy.

Additionally, as a metabolite produced by GM, H_2_ gas plays an important role in maintaining tumor resistance. Current studies have demonstrated that H_2_ therapy is effective in treating various cancers with no significant side effects. Moreover, recent research has explored the potential for combining H_2_ therapy with conventional treatments such as chemotherapy and radiotherapy, demonstrating improved efficacy and reduced side effects [254]. However, despite its promising potential as an adjunctive cancer therapy, H_2_ gas alone may not achieve sufficient therapeutic effects. At this stage, H_2_ therapy should not replace traditional treatments such as chemotherapy or radiotherapy. Future research should focus on the combined application of H_2_ gas with other emerging therapies to fully harness its potential.

## 6 Clinical Applications of Molecular H_2_

With the successful demonstration of molecular H_2_’s therapeutic and preventive effects on various diseases in animal models, the clinical application of H_2_ has also gained importance, with several studies in recent years showing that H_2_ has demonstrated good therapeutic efficacy and safety in clinical settings. In this article, we reviewed the molecular mechanisms through which H_2_ alleviates intestinal diseases in animal models and discussed its impact on other diseases mediated via the GM [225]. Here, we will briefly summarize the clinical applications of H_2_ in treating gastrointestinal diseases and provide an overview of clinical trials involving its use in respiratory, neurological, cardiovascular, chronic diseases, and cancer.

### 6.1 Clinical Applications of Molecular H_2_ in the Treatment of Gastrointestinal Diseases or Through Modulation of GM

It should be acknowledged that there are currently few clinical trials of H_2_ for the treatment of intestinal disorders, but it is worth noting that the measurement of exhaled H_2_ and CH_4_ gases following the ingestion of readily metabolizable carbohydrates in humans has emerged as an important noninvasive breath test to aid in the diagnosis of SIBO [255, 256]. This is the most widespread clinical application of H_2_ to assist in the treatment of intestinal disorders. Furthermore, noninvasive detection of head and neck squamous cell carcinoma [257], gestational diabetes mellitus [258], heart failure [259], and autism (ASD) [260], based on the ratio of exhaled H_2_ to CH_4_, has also shown potential, because SIBO is associated with the development of some diseases, and alterations in the composition of the GM of the patient result in changes in the amount of exhaled H_2_ gas. Additionally, there are several clinical uses of H_2_ in the treatment of other diseases that have been demonstrated to work by acting on the GM, such as Xie et al. [261] who found that H_2_ could alter the composition of the GM in people with opioid addiction and thus act on the gut–brain axis to improve the symptoms of depression and anxiety in people addicted to opioids. In a randomized double‐blind controlled clinical study, Liang et al. [262] found that HRS could improve metabolism by modulating GM in patients with impaired fasting glucose (Table 4).

**TABLE 4:** The clinical applications of molecular hydrogen in the treatment of gastrointestinal diseases, respiratory disorders, neurological conditions, cardiovascular diseases, chronic illnesses, and cancer are outlined.^a^

Diseases	Route	Number of patients and protocol	Effects	Limitations	Trial phase	Date posted	Clinical trial reference
Molecular hydrogen to treat gastrointestinal diseases or act on gut microbiota							
CRC	Hydrogen inhalation	55, Duration 3 h/day/3 months	PD‑1+ CD8+↓, Mitochondria↑, PD‑1‐ CD8+↑	Adherence issues	Phase II	January 2019	[206]
IBS	Oral lactulose	263, Drinking a combined lactulose nutrient drink every 15 min for 4h	Lactulose hydrogen breath test	Noncontrolled study	Phase II	September 2022	[256]
HNSCC	Produced in the intestines	135, Breath samples taken after 6 h of fasting	H_2_BT	/	Phase II	August 2020	[257]
GDM	Produced in the intestines	320, fasting for 12 h	H_2_BT	Single‐center study	Phase II	June 2021	[258]
HF	Oral lactulose	102, Drinking a combined lactulose nutrient drink	Lactulose hydrogen breath test	Noncontrolled study	Phase II	October 2018	[259]
ASD	Produced in the intestines	1550, Exhaled gases were collected both after a 12‐h fast and following glucose intake	H_2_BT	/	Phase III	August 2017	[260]
Opioid addiction	Hydrogen inhalation	108, Duration 2 h/day/3 months	Regulates gut microbiota	No stratification	Phase II	August 2024	[261]
IFG	HRW, oral	73, Drink 1000 mL of HRW per day for 8 weeks	Regulates gut microbiota	No stratification	Phase II	June 2023	[262]
Molecular hydrogen therapy for respiratory diseases							
Asthma and COPD	Inhalation of 2.4% H_2_	20, 2.4% hydrogen containing steam mixed gas was inhaled once for 45 min	Inhibits IL‐4, IL‐6, and IL‐1β	Small sample size	Phase I	May 2020	[263]
AECOPD	Inhalation of hydrogen–oxygen mixture	108, hydrogen–oxygen mixture therapy (v/v = 2:1), flow rate 3.0 L/min, 6–8 h/d.	Improvement in symptoms such as dyspnea, cough, and sputum production in patients	No stratification	Phase II	May 2021	NCT04000451
COVID	Inhalation of hydrogen–oxygen mixture	88, hydrogen–oxygen mixture therapy (v/v = 2:1), flow rate 3.0 L/min; 6–8 h/d	Increased SpO_2_	No stratification	Phase I	June 2023	NCT04378712
Tracheal Stenosis	Inhalation of hydrogen–oxygen mixture	35, Air, H₂–O₂, and O₂ inhalation was administered in four consecutive breathing steps: air for 15 min, H₂–O₂ (6 L/min, H₂:O₂ = 2:1) for 15 min, oxygen (3 L/min) for 15 min, and H₂–O₂ for 120 min	Reduce the inspiratory effort	No stratification	Phase I	September 2018	NCT04378712
Long‐COVID	HRW, oral	32, 500 mL HRW, twice a day for14 days	Effective strategies to reduce fatigue and improve cardiorespiratory endurance, musculoskeletal function, and sleep quality	Small sample size	Phase I	May 2024	[266]
Molecular hydrogen therapy for neurological diseases							
PD	Inhalation of hydrogen–oxygen mixture	20, Participants inhaled 6.5 (0.1) vol% H_2_ in 2 L/min of mixed air for 16 weeks, twice a day for 1 h each session	No beneficial effects were found, but safety was assured	Small sample size	Phase I	July 2021	UMIN000039217
Cerebral infarction	Injections of HRS	34, HRS intravenously for 7 days	Reduced patient MRI index	Small sample size	Phase I	June 2011	[268]
Acute cerebral ischemia	Injections of HRS	38, Intravenous HRS at 200 mL/h twice daily (every 12 h)	No beneficial effects were found, but safety was assured.	Open‐label study	Phase I	June 2013	[269]
SAH	Injections of HRS	450, Intravenous hydrogen‐rich fluid infusion (200 mL) twice daily for 14 days	Results not yet published	No stratification	Phase II	September 2014	UMIN000014696
Cerebral infarction	Inhalation of 3% H_2_	50, Inhalation of 3% H_2_ (1 h twice daily) for 7 days	Improve NIHSS score	Limited follow‐up duration	Phase I	November 2017	[271]
AD	Inhalation of 3% H_2_	19, Inhalation of 3% H_2_ (1 h twice daily) for 6 months	Improvement of neuronal integrity in the hippocampus	Small sample size	Phase I	March 2023	[272]
Molecular hydrogen therapy for cardiovascular system diseases							
PCAS	Inhalation of 2% H_2_	5, Inhalation of 2% H_2_ for 18 h	No adverse effects identified	Small sample size	Phase I	July 2016	[273]
Patients undergoing CPB	Inhalation of 1.5–2% H_2_	24, Inhalation of 1.5–2% H_2_ throughout procedure	Increased ATP and reduced oxidative stress	Small sample size	Phase I	April 2023	[274]
Potential metabolic syndrome	HRW, oral	20, of HRW for 10 weeks (0.9–1.0 L/day)	Decreased LDL‐C and apoB levels and increased SOD activity	Small sample size	Phase I	July 2013	[275]
Cardiogenic OHCA	Inhalation of 2% H_2_	72, Inhalation of 2% H_2_ for 18 h	Combined TTM improves neurological prognosis after cardiogenic OHCA	No stratification	Phase II	August 2024	jRCTs031180352
Molecular hydrogen therapy for chronic diseases							
T2DM	HRW, oral	36, Drinking 900 mL/d of HRW for 8 weeks	Reduces LDL and cholesterol	Noncontrolled study	Phase I	March 2008	[277]
Dry eye disease	Hydrogen production capsule	10, 10 capsules of SUPER H2	Increased tear secretion	Small sample size	Phase I	March 2021	UMIN000037169
NAFLD	Inhalation of 66% H_2_	43, inhalation hydrogen–oxygen (v/v = 2:1) mixture therapy (3 L/min) for 1 h/d	Lowering blood lipids and reducing inflammation	Noncontrolled study	Phase I	June 2022	ChiCTR‐IIR‐16009114
NAFLD	HRW, oral	12, Per day of HRW for 28 days	Decreased liver fat content and a reduction in serum AST levels	Small sample size	Phase I	April 2019	NCT03625362
Visceral fat and skin spots	Hydrogen‐rich water bath	4, The subjects underwent a daily 10‐min warm (41°C) water bath with dissolved hydrogen (300–310 µg/L, < 10 µg/L for normal water) for 1–6 months	Reduces LDL and cholesterol, and reduces skin blemishes	Small sample size	Phase I	April 2019	[280]
Psoriasis and Parapsoriasis en plaques	Hydrogen‐rich water bath	47, Hydrogen‐water bathing was administered through the skin by immersing the whole body in hydrogen water twice a week, with a 3‐day interval	Reduction in the area of damaged skin and relief of itching	Noncontrolled study	Phase I	May 2018	ChiCTR‐ONC‐17013055
Molecular hydrogen therapy for chronic diseases							
Psoriasis	Drop (1 ppm H_2_‐saline); inhalation of 3% H_2_; drinking of HRW	3, Three methods were used to administer H_2_	Individual patients had reduced levels of IL‐6 and IL‐17	Small sample size	Phase I	August 2015	[96]
Inflammatory	HRW bath with nano‐sized bubbles	15, Daily bathing for up to 2–25 months	Reduces inflammation in patients and improves antioxidant capacity in healthy individuals	Small sample size	Phase I	July 2022	[282]
Inflammatory	Oral solid hydrogen capsules	30, Low dose group: Medium dose group: High dose group (1:3:6 capsule per day) for 28 days	Anti‐inflammatory and antioxidant	Small sample size	Phase I	September 2024	[283]
Overweight	HRW, oral	5, One liter of HRW day	Attenuate glutamate‐ and GABA‐dependent hypothalamic appetite stimulation, leading to hunger suppression and weight loss	Small sample size	Phase I	February 2023	NCT06722326
Molecular hydrogen therapy for cancer							
Stage III and IV cancer patients	Inhalation of 66% H_2_	82, Inhalation hydrogen–oxygen (v/v = 2:1) mixture therapy (flow rate of 3000 mL/min) for inhalation time >3 h per day for at least >3 months	Treating different cancers has different effects, but can improve the quality of life of cancer patients	/	Phase I	July 2019	[284]
Non‐small cell lung cancer	Inhalation of 66% H_2_	20, Inhalation of a mixture of hydrogen (66.7%) and oxygen (33.3%) at a flow rate of 3 L/min for 4 h per day for 2 weeks	Decreased number of depleted and senescent cytotoxic T cells and increased killer Vδ1 cells	Small sample size	Phase I	October 2020	NCT03818347
Non‐small cell lung cancer	Inhalation of 66% H_2_	58, Patients treated with hydrogen were divided into four groups: H_2_‐only, H_2_ + chemotherapy, H_2_ + targeted therapy, and H_2_ + immunotherapy groups	Controlling cancer progression to reduce drug therapy side effects	Single‐center study	Phase I	April 2020	NCT03818347
Bone marrow damage in cancer patients	Inhalation of 3% H_2_	23, Inhalation of 3% H_2_ (flow rate of 4 L/min for 30 min/day)	Improves bone marrow damage in patients by increasing RBC and PLT counts	Small sample size	Phase I	July 2021	UMIN000035864

^a^ This includes an overview of the diseases under investigation, methods of administration, the number of patients, treatment protocols, outcomes, limitations, stages of experimentation, the date of the initial trial submission, and clinical trial references (Numbers correspond to references in this article, and other numbers represent US Clinical Trial Numbers, EU Clinical Trial Numbers, Japanese Clinical Trial Numbers, and Chinese Clinical Trial Numbers.).

*Abbreviations*: AD, Alzheimer's disease; ASD, autism spectrum disorders; COPD, chronic obstructive pulmonary disease; CPB, cardiopulmonary bypass; GDM, gestational diabetes mellitus; HF, heart failure; HNSCC, head and neck squamous cell carcinoma; IBS, irritable bowel syndrome; IFG, impaired fasting glucose; NAFLD, nonalcoholic fatty liver disease; OHCA, out‐of‐hospital cardiac arrest; PCAS, post‐CABG angina syndrome; PD, Parkinson's disease; SAH, subarachnoid hemorrhage; T2DM, type 2 diabetes mellitus.

### 6.2 Clinical Applications of Molecular H_2_ in Respiratory Diseases

H_2_ is particularly effective in treating respiratory diseases, as it is primarily administered by inhalation, and clinical studies have demonstrated the potential of H_2_ for diseases such as asthma, COPD, and COVID‐19. Wang et al. [263] found that a single 45‐min inhalation of 2.4% H_2_‐containing steam mixed gas reduced airway inflammation in both asthmatics and COPD patients in a prospective study of 10 asthmatics and COPD patients. Niu et al. [264] assessed glycolytic and mitochondrial oxidative phosphorylation activity in asthmatics and a mouse model of allergic airway inflammation, showing that H_2_ increased ATP production as well as the activity of mitochondrial respiratory chain complexes I and III. Zheng et al. [265] conducted a prospective clinical trial to evaluate whether HO mixtures are superior to oxygen in improving symptoms in patients with acute exacerbation of COPD (AECOPD). The results showed a significant reduction in cough assessment test scores in the H_2_/oxygen group, with no adverse events reported, indicating that H_2_/oxygen therapy was more effective and safe compared with standard oxygen therapy [265]. Similarly, Zeng et al. [101] treated 33 patients with H_2_/oxygen therapy and found that it improved dyspnea and disease progression in COVID‐19 patients, resulting in higher SpO_2_ levels and shorter hospital stays. Tan et al. [266] conducted a randomized, single‐blind, placebo‐controlled trial with 32 participants and found that although H_2_‐enriched water did not improve dyspnea in patients with long‐COVID compared with acute COVID‐19, it alleviated fatigue and improved cardiorespiratory endurance, musculoskeletal function, and sleep quality (Table 4).

### 6.3 Clinical Applications of Molecular H_2_ in Neurological Diseases

Animal experiments have demonstrated that H_2_ plays a beneficial role in treating neurological disorders, while human trials suggest its potential therapeutic value for neurodegenerative conditions. Yoritaka et al. [267] conducted a randomized, double‐blind, placebo‐controlled pilot study to investigate the ameliorative effects of inhaled H_2_ on PD, but their findings indicated that although inhalation of H_2_ was safe, it had no significant beneficial effect on PD patients. Although molecular H_2_ demonstrates limited therapeutic efficacy in PD, its antioxidant effects and mitochondrial function modulation in acute brain injury and neurodegenerative diseases remain scientifically noteworthy. Ono et al. [268] recruited 34 patients with cerebral infarction due to atherosclerotic disease, dividing them into two groups: one with 26 patients receiving edaravone alone, and the other with eight patients receiving a combination of edaravone and HRS. Surprisingly, the combination group showed a more pronounced improvement in MRI markers [268]. Nagata et al. [269] recruited 38 patients hospitalized for acute ischemic stroke in an open‐label, prospective, nonrandomized study to assess the safety of intravenous H_2_‐enriched glucose‐electrolyte solution combined with t‐PA. The results indicated that the therapy was relatively safe for patients with acute cerebral infarction, though the study lacked a separate H_2_‐only group due to insufficient patient numbers, and one patient experienced diarrhea, which could not be definitively linked to the treatment [269]. In a 2014 publication by Takeuchi et al. [270], they announced that they had initiated a double‐blind randomized controlled trial (RCT) in a large study population, enrolling 450 patients with high levels of SAH, in order to evaluate the efficacy of H_2_ therapy in the treatment of early brain injury, delayed cerebral ischemia and vasospasm, but unfortunately, the results of this clinical study have not been published so far. Following the discovery of the synergistic effects of H_2_ with other therapies, Ono et al. [271] later recruited 50 patients with acute cerebral infarction to study the effects of H_2_ alone. The patients were divided into two groups, one receiving H_2_ treatment and the other receiving gas without H_2_, and the results showed that H_2_ treatment stabilized vital signs and improved patients' ability to perform daily activities, with no adverse reactions observed. No adverse effects were found during the experiment, and safety was guaranteed [271]. In 2023, Ono et al. [272] published another article demonstrating that inhalation of 3% H_2_ alone for 1 h for 6 months provided symptomatic relief and improvement in patients with AD (Table 4).

### 6.4 Clinical Applications of Molecular H_2_ in Cardiovascular System Diseases

At present, the ameliorative effect of H_2_ on cardiovascular disease in humans still needs to be confirmed by large‐scale study populations. However, some clinical studies have already demonstrated the high safety and feasibility of H_2_ in treating cardiovascular disease. The first human pilot study of H_2_ for post‐CA syndrome was conducted in 2016, showing that inhalation of H_2_ led to a favorable neurological prognosis. However, the study was limited by a small sample size and the short duration of H_2_ inhalation due to the capacity limitations of the gas cylinders [273]. In another study, 24 patients undergoing cardiopulmonary bypass (CPB) surgery were randomized into two groups: 12 patients received inhaled H_2_ at a concentration of 1.5–2.0%, while the control group did not receive H_2_, and the results showed that H_2_‐treated patients had improved erythrocyte function and reduced oxidative stress [274]. Song et al. [275] found beneficial lipid‐lowering effects of H_2_ in animal studies, and thus they included 20 patients with potential metabolic syndrome to investigate the ameliorative effects of H_2_‐enriched water in humans, showing that supplementation with H_2_‐enriched water appeared to reduce serum low‐density lipoprotein cholesterol (LDL‐C) and apolipoprotein B (apoB) levels, and to improve the function of dyslipidemia‐impaired high‐density lipoprotein cholesterol, reducing oxidative stress. This means that H_2_ may play a beneficial role in the prevention of potential metabolic syndrome [275]. In a recent posthoc analysis of a RCT, Tamura et al. [276] found that the combination of H_2_ inhalation and Targeted Temperature Management (TTM) (TTM32–TTM34) improved neurological prognosis after cardiogenic out‐of‐hospital cardiac arrest (OHCA) compared with TTM only (TTM32–TTM34) at 32–34°C. However, the limitations of this study also need to be taken into account; this was a posthoc analysis with sample size, and the results of this study could be used as a reference to design a larger sample size to be explored in subsequent studies (Table 4).

### 6.5 Clinical Applications of Molecular H_2_ in Chronic Diseases

As early as 2008, 1 year after the anti‐inflammatory and antioxidant effects of H_2_ were first identified, a randomized, double‐blind, placebo‐controlled crossover trial involving 30 patients with type 2 diabetes mellitus (T2DM) demonstrated the beneficial effects of HRW consumption in preventing T2DM and insulin resistance [277]. Another small exploratory study revealed the protective effect of H_2_ on the lacrimal gland. This study, which involved both mice and 10 human subjects, showed that H2‐producing supplements significantly increased the concentration of exhaled H_2_ and improved tear stability and symptoms of dry eye [278]. Tao et al. [279] conducted a 13‐week clinical trial of H_2_/oxygen inhalation in 43 subjects with NAFLD. Their findings showed that H_2_/oxygen inhalation improved blood lipid profiles and liver enzyme levels, with significant improvements in hepatic fat content in moderate‐to‐severe cases as detected by ultrasound and CT scanning [279]. In another study, Asada et al. [280] selected two men and two women to investigate the effects of HRW on oxidative stress‐related skin issues and lipid metabolism markers, as well as to determine whether dissolved H_2_ remained effective after boiling. The results indicated that HRW baths improved visceral fat and skin spots and that dissolved H_2_ showed resistance to high temperatures [280]. Zhu et al. [281] found that HRW baths had a therapeutic effect on psoriasis, with patients receiving HRW baths experiencing a reduction in the size of their psoriasis and a reduction in itching. In another clinical study on psoriasis treatment, H_2_ was administered via HRS injection, HRW ingestion, and inhalation of 3% H_2_. The results suggested a reduction in IL‐6 and IL‐17 levels in individual patients following H_2_ treatment [96]. Nanobubble HRW baths provide better solubility of H_2_ in water than HRW baths alone and reduce H_2_ escape to a certain extent. Tanaka et al. [282] found that nanobubble HRW baths not only alleviated the inflammatory symptoms and skin appearance of patients with connective tissue diseases but also improved the serum antioxidant capacity of healthy patients. In 2022, a clinical study demonstrated the anti‐inflammatory and antioxidant effects of orally administered solid H_2_ capsules in individuals with chronic inflammation, highlighting the potential long‐term health benefits of H_2_ [283] (Table 4).

### 6.6 Clinical Applications of Molecular H_2_ in Cancer

Recent years have witnessed groundbreaking advancements in the application research of H₂ within cancer therapeutics. Chen et al. [284] conducted a prospective follow‐up study involving 82 patients with stage III and IV cancers receiving H_2_ inhalation therapy. They found that H_2_ inhalation improved the quality of life and helped control cancer progression, with effects varying across different cancers. The best outcomes were observed in lung cancer patients, whereas pancreatic and gynecological cancers showed the least improvement [284]. Chen et al. [92] included 20 patients with non‐small cell lung cancer to study the ameliorative effects of H_2_ and found that the superior therapeutic effect of H_2_ in lung cancer patients was mainly through the improvement of immune senescence in lymphocyte populations. Beyond its direct antitumor effects, molecular H_2_’s capacity to mitigate treatment‐related adverse effects provides a strategic advantage for its integration into comprehensive cancer care protocols. In another clinical trial the following year, Chen et al. [254] recruited 58 adult patients to investigate the effects of H_2_ inhalation alone versus H_2_ in combination with chemotherapy, targeted therapy, and immunotherapy, respectively, for the treatment of advanced non‐small‐cell lung cancer. Sixteen months of follow‐up found that progression‐free survival in the control group was lower than that in the H_2_ inhalation group alone, and significantly lower than that in the other three combination therapy groups. In this study, not only the therapeutic effect of H_2_ alone but also the synergistic effect of H_2_ in combination with other therapies was highlighted [254]. In a retrospective observational study, Hirano et al. [285] found that H_2_ inhalation showed promising results in cancer treatment and also ameliorated bone marrow damage induced by intensity modulated radiation therapy (IMRT) in cancer patients, without compromising the antitumor effects of IMRT, thereby underscoring its potential in adjuvant therapy. These findings provide multilayered evidence for the integration of H_2_ in comprehensive cancer management. The clinical value of molecular H_2_ has become increasingly prominent across multiple systemic diseases mentioned above. Future research necessitates sustained breakthroughs in three dimensions: in‐depth exploration of mechanisms (e.g., epigenetic regulation), optimization of intervention strategies (e.g., precision delivery systems), and interdisciplinary integration (e.g., H_2_ medicine with artificial intelligence (Table 4).

## 7 Conclusions and Prospects

Molecular H_2_ has gained widespread attention in the medical field, with studies showing its multiple properties, including anti‐inflammatory, selective antioxidant, antiapoptotic, energy metabolism regulation, and immune modulation, making it widely used in the treatment of various diseases. However, the mechanisms and therapeutic efficacy of molecular H_2_ in different patients still need further investigation. Molecular H_2_ targets core pathways such as NF‐κB, MAPK, and the NLRP3 inflammasome to synergistically regulate and suppress inflammation. However, research by An et al. [53] found that estrogen can enhance H_2_’s inhibitory effect on p‐NF‐κB, suggesting that gender differences may influence the anti‐inflammatory effects of molecular H_2_. The antioxidant effect of molecular H_2_ is mainly achieved by selectively scavenging toxic free radicals and activating endogenous antioxidant systems. Elderly patients are more sensitive to H_2_ therapy due to mitochondrial dysfunction and a decreased ability to eliminate free radicals, which may result in the accumulation of chronic inflammation and oxidative stress. This also explains the significant intervention effects of molecular H_2_ in treating age‐related diseases, such as atherosclerosis and Alzheimer's disease. Through the Nrf2 pathway, molecular H_2_ induces the expression of antioxidant enzymes (such as SOD, CAT, and GPx), enhancing cell tolerance to oxidative stress. Therefore, the polymorphisms of genes such as Nrf2 may affect the efficacy of molecular H_2_. In the future, genetic testing could be used to identify H_2_‐sensitive genetic markers to optimize treatment plans for specific populations. Although the mechanisms of molecular H_2_ have been somewhat confirmed, its protein and gene‐level targets remain unclear. Yu et al.’s research team [286], through proteomics and genomics analysis, identified 199 proteins that are considered highly relevant to the mechanism by which H_2_ improves gut barrier function in sepsis patients. Additionally, Ohsawa et al. [287] found through metabolomics analysis that transient H_2_ exposure can inhibit energy metabolism‐related pathways (such as the reduction of glutathione levels) and trigger protective stress mechanisms. Chen et al. [288], based on UPLC–QTOF/MS metabolomics, revealed the regulatory effects of H_2_ on the metabolic pathways in mice with ischemic stroke. These studies suggest that, in the future, multiomics joint analysis can be used to explore the interaction between molecular H_2_ and biological systems, uncover new targets, and enable precision therapy. While potential therapeutic mechanisms still need further exploration, existing research evidence indicates that H_2_ therapy has become an efficient tool for treating various diseases. To date, there has been only one reported case of diarrhea, which may be an isolated incident. However, whether H_2_ therapy is truly perfect and whether there are undetectable potential adverse reactions still needs further verification.

H_2_ exerts its influence on the treatment of intestinal diseases through different mechanisms. For instance, it regulates the production of SCFAs mediated by GM to ameliorate intestinal inflammation, suppresses inflammatory and apoptosis signaling pathways to inhibit colitis progression, and modulates immunity for effective management of colon cancer. However, despite this significant progress, there remains a relative scarcity of research on the molecular mechanisms underlying H_2_ as a signaling molecule for treating intestinal diseases, most of the current research on H_2_ for the treatment of intestinal diseases focuses on basic research on the use of H_2_ therapy alone, with only a few cases exploring the effects of H_2_ therapy in combination with existing drugs for the treatment of intestinal diseases. According to the current combined therapeutic effect of H_2_ and clinical drugs, the safety of H_2_ in the treatment of intestinal diseases can be ensured and it shows potentiation, but the mechanism of H_2_ in the treatment of intestinal diseases is still to be studied in depth. It is also worth noting the close relationship between the intestinal flora and H_2_. As we have said before, many diseases are associated with changes in the GM. In the previous section, we summarized the fact that many diseases are associated with changes in the GM and that treatment of the disease is often followed by a restoration of the diversity of the GM. In recent years, the “gut–brain axis,” “gut–liver axis,” and “gut–kidney axis” have attracted significant attention, particularly the “gut–brain axis.” Changes in the diversity of the GM are accompanied by neurodevelopmental changes throughout the human lifespan, with many bi‐directional communication pathways between the GM and the brain, including immune‐regulatory responses, neuronal innervation, and microbial metabolite signaling. The intestinal flora is symbiotic with H_2_‐producing and H_2_‐consuming bacteria, and it is known that exogenous H_2_ produces changes in the composition and diversity of GM, but the duration of such changes is unknown, and whether the balance of H_2_‐producing and H_2_‐consuming bacteria undergoes new changes when exogenous H_2_ is discontinued needs to be studied in greater depth. In conclusion, the close relationship between GM and H_2_ should not be ignored, and the modulation of GM composition and function by H_2_ and the modulation of GM metabolites by H_2_ require in‐depth study, which will help to discover new therapeutic targets and mechanisms of action of H_2_.

H_2_ has shown good therapeutic effects through different delivery modes. However, the clinical utilization of H_2_ presents several challenges. Although there are many means of H_2_ delivery in animal experiments, the delivery modes in clinical applications are relatively homogeneous, and more clinical trials are needed for validation, especially for the clinical promotion of nanomaterial‐assisted delivery of H_2_. There are still some drawbacks to be addressed with the several modes of H_2_ delivery commonly used in clinical practice. First, managing the equipment for preparing, storing, transferring, and distributing inhaled H_2_ requires meticulous care to prevent potential explosions. Although the current clinical H_2_ concentration is between 2 and 4%, which is generally safe from explosion, safety considerations and cost make the clinical use of higher concentrations difficult. Some studies indicate that the efficacy of H_2_ may be dose dependent, with higher concentrations possibly providing better treatment outcomes for certain conditions. Second, oral administration of HRW lacks targeted delivery to specific lesions. Last, issues related to the timing, method, and dosage of HRW injections need resolution.

In addition to the challenges of drug delivery methods, the clinical promotion of molecular H_2_ also faces several key issues: (1) *Limited clinical trials*: Most clinical trials on H_2_ are small, single‐center studies, lacking large‐scale, multicenter, RCTs to verify long‐term efficacy. Additionally, there are significant variations in the doses and concentrations of molecular H_2_ used (e.g., inhaled H_2_ concentrations range from 2 to 66%), and treatment protocols have not been adjusted based on disease stages (such as early/late‐stage cancer) or comorbidities (such as diabetic nephropathy). Future research needs to explore the timing, dose‐response relationship, and how H_2_ can be combined with other therapies. This requires larger sample sizes and longer follow‐up periods for validation. (2) *Safety concerns with combination therapies*: The safety of combining H_2_ with other medical gases, as well as how different populations accept and adjust H_2_ concentrations, requires further study. Currently, H_2_ is mainly combined with oxygen, and only a few studies have investigated using H_2_ with other gases (e.g., nitrogen–oxygen–H_2_ mixtures). The impact of H_2_ on specific diseases, particularly those related to genetic background or sex hormones, also needs further validation. (3) *Adjunctive therapeutic effects*: More research is needed on the adjunctive therapeutic effects of H_2_. Some studies have shown that the high biocompatibility of molecular H_2_ enhances the effects of existing therapies without interference, particularly in reducing the side effects of chemotherapy and radiotherapy. However, more animal and clinical trials are necessary to verify these findings. (4) *Precision H_2_ therapy for different populations*: Despite H_2_’s broad intervention effects, its efficacy is influenced by factors such as age, gender, and genetic background. However, clinical studies often lack patient stratification, and personalized treatment remains a significant challenge. (5) *Standardized regulatory framework*: A standardized regulatory framework for H_2_ clinical trials and long‐term safety assessments is needed. The appropriate concentrations of H_2_ for patient inhalation, drinking HRW, or injecting HRS should be clearly defined. The therapeutic effects of high‐concentration H_2_ should be explored within a reasonable range. Additionally, distinctions between chronic and acute diseases should be made, and differentiated regulatory indicators should be established.

In recent years, nanomedicine has developed rapidly, and the efficacy of nanomaterials in addressing H_2_ gas diffusion and targeted therapy has been well established. Numerous studies have demonstrated that H_2_ gas encapsulated in nanomaterials exhibits significant therapeutic effects in treating various diseases. However, its application in the treatment of gastrointestinal diseases remains limited. While nanomaterials can help reduce H_2_ gas leakage and assist in targeted H_2_ therapy, their long‐term safety, potential for abnormal immune responses, and biodegradability still require further validation in preclinical and clinical trials. Future efforts should focus on the development of nontoxic, biodegradable nanomaterials to aid H_2_ delivery. H_2_ medicine is an emerging scientific field that has made remarkable progress in recent years. Further elucidation of its underlying mechanisms will provide valuable insights for clinical applications. In the future, H_2_ medicine and nanohydrogen medical systems are expected to significantly improve, offering new opportunities for the diagnosis, treatment, and prevention of human diseases.

## Author Contributions

Xiaohong Pan, Qianqian Lu, and Yuming Zhou contributed to reviewing, editing, and supervising the manuscript. Jiayi Jin was involved in writing the first draft as well as creating all the illustrations and tables. Lijun Yue, Maoru Du, Feng Geng, and Xue Gao were responsible for reviewing the literature. All authors have read and approved the final manuscript.

## Conflicts of Interest

The authors declare no conflicts of interest.

## Ethics Statement

The authors have nothing to report.

## Data Availability

The authors have nothing to report.
